# The Cofilin Phosphatase Slingshot Homolog 1 (SSH1) Links NOD1 Signaling to Actin Remodeling

**DOI:** 10.1371/journal.ppat.1004351

**Published:** 2014-09-04

**Authors:** Harald Bielig, Katja Lautz, Peter R. Braun, Maureen Menning, Nikolaus Machuy, Christine Brügmann, Sandra Barisic, Stephan A. Eisler, Maria Andree, Birte Zurek, Hamid Kashkar, Philippe J. Sansonetti, Angelika Hausser, Thomas F. Meyer, Thomas A. Kufer

**Affiliations:** 1 Institute for Medical Microbiology, Immunology and Hygiene, Cologne, Germany; 2 Department of Molecular Biology, Max Planck Institute for Infection Biology, Berlin, Germany; 3 Steinbeis-Innovationszentrum Center for Systems Biomedicine, Falkensee, Germany; 4 Institute of Cell Biology and Immunology, University of Stuttgart, Stuttgart, Germany; 5 Unité de Pathogénie Microbienne Moléculaire, Institut Pasteur, Paris, France; 6 INSERM U786, Institut Pasteur, Paris, France; 7 Microbiologie et Maladies Infectieuses, Collège de France, Paris, France; 8 University of Hohenheim, Institute of Nutritional Medicine, Stuttgart, Germany; University of California, Davis, United States of America

## Abstract

NOD1 is an intracellular pathogen recognition receptor that contributes to anti-bacterial innate immune responses, adaptive immunity and tissue homeostasis. NOD1-induced signaling relies on actin remodeling, however, the details of the connection of NOD1 and the actin cytoskeleton remained elusive. Here, we identified in a druggable-genome wide siRNA screen the cofilin phosphatase SSH1 as a specific and essential component of the NOD1 pathway. We show that depletion of SSH1 impaired pathogen induced NOD1 signaling evident from diminished NF-κB activation and cytokine release. Chemical inhibition of actin polymerization using cytochalasin D rescued the loss of SSH1. We further demonstrate that NOD1 directly interacted with SSH1 at F-actin rich sites. Finally, we show that enhanced cofilin activity is intimately linked to NOD1 signaling. Our data thus provide evidence that NOD1 requires the SSH1/cofilin network for signaling and to detect bacterial induced changes in actin dynamics leading to NF-κB activation and innate immune responses.

## Author Summary

NOD1 was one of the first NLR-family members shown to act as an important intracellular pattern-recognition molecule mediating antimicrobial activities in mammals. It has been demonstrated that perturbation of F-actin and RhoGTPase activity affects NOD1 and NOD2 signaling, however, the effectors of this process remained elusive. By using a multilayered high-throughput druggable genome wide siRNA screening approach to discover novel components specific for the NOD1 pathway, we identified the cofilin phosphatase SSH1, which acts downstream of RhoA-ROCK, as key regulator of NOD1 signaling. We show that SSH1 forms a complex with NOD1 at F-actin rich sites in human cells and is needed for NOD1-mediated responses towards TriDAP exposure and *Shigella flexneri* infection. Functionally this is achieved by SSH1-mediated activation of cofilin. Our findings reveal a previously unrecognized role for SSH1 in NOD1 signaling and provide a plausible unifying mechanistic explanation of how perturbations of the actin cytoskeleton can induce NOD1-mediated inflammatory responses.

## Introduction

Effective immune defense in mammals relies on the detection of conserved pathogen structures by pattern recognition receptors (PRRs) of the innate immune system to prime immune responses [1].

Several PRRs have been identified and extensively studied in the last decade. In particular, members of the NOD-like receptor (NLR)-family gained attention due to their intracellular localization [2], [3]. One of the first NLRs shown to act as a PRR is NOD1. NOD1 is an intracellular protein that can be activated by diaminopimelic acid-containing peptides derived from bacterial peptidoglycan and acts as a sensor for invasive bacteria such as *Shigella flexneri* [4]–[6].

A wealth of data suggest that NOD1 is an important PRR for a variety of bacteria in mammals, which also contributes to systemic activation of neutrophils, induction of adaptive immunity and immune tissue homeostasis (reviewed in [2], [3]). Upon activation, NOD1 forms a complex with the receptor-interacting serine/threonine-protein kinase 2 (RIP2), which results in the activation of NF-κB and mitogen-activated protein kinases (MAPK) signaling pathways [2], [3]. Several components of the pathway downstream of NOD1 have been identified. For example, the NOD1 binding partner RIP2 mediates activation of the TGF-β-associated kinase 1 (TAK1) complex which is induced by ubiquitylation of RIP2 through ubiquitin ligases including the X-linked inhibitor of apoptosis protein (XIAP) [7] and the cellular inhibitor of apoptosis protein-1 and -2 (cIAP1, and cIAP2) [8]. NOD1 is found at the plasma membrane where it co-localizes with F-actin. This localization was suggested to be a prerequisite for signaling because affecting actin polymerization changes NOD1 signaling [9]. Furthermore, the *Salmonella* effector SopE activates NOD1, involving changes in small Rho GTPase activity [10]. Additionally, the RhoA guanine nucleotide exchange factor H1 (GEF-H1) was linked to NOD1 activation [11]. Of note, the NOD1 related protein NOD2 is also regulated by the small GTPase Rac1 [12], [13] and localizes at the plasma membrane at cortical F-actin structures, similar to NOD1 [9], [13], [14]. Together this indicates an intimate connection of NOD1 and NOD2 signaling with the actin cytoskeleton, although the mechanistic details remain largely elusive. Cellular actin dynamics are strictly controlled by the action of nucleation factors such as Arp2/3, which bind to the sides of pre-existing filaments and promote the growth of new filaments at these sites. Actin binding proteins belonging to the actin depolymerization factors (ADF)/cofilin family control the disassembly of actin filaments by severing F-actin filaments, thereby generating new sites of actin polymerization. In addition, there is evidence that cofilin depolymerizes F-actin to provide new G-actin molecules for polymerization. Cofilin activity itself is tightly controlled by LIMK1 and LIMK2, which phosphorylate cofilin at serine 3 whereby its activity is blocked. Accordingly, dephosphorylation by the phosphatase slingshot homolog 1 (SSH1) reactivates cofilin (reviewed in [15]).

Here we identify the cofilin phosphatase SSH1 as an essential component of the human NOD1 signaling pathway and show that SSH1 links NOD1 activation to cofilin-mediated changes in actin remodeling.

## Results

### A high-throughput siRNA-screen identifies SSH1 as an essential component of NOD1-mediated NF-κB signaling

To identify novel factors involved in NOD1-mediated NF-κB activation, we adapted a cell based NF-κB-luciferase reporter gene assay in HEK293T cells [16] for high-throughput (HT) small interfering RNA (siRNA) screening (Figures S1A and B).

**Figure 1:**
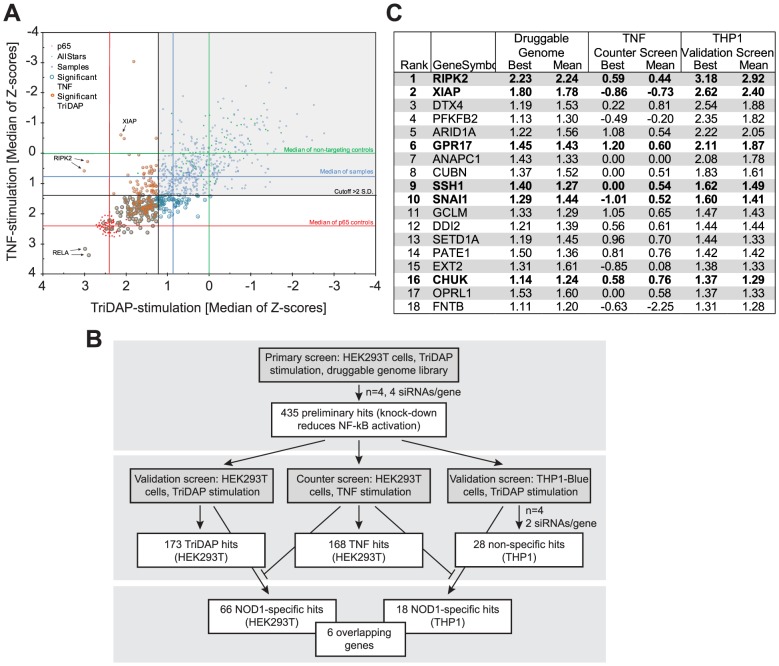
HTS siRNA-screening identifies SSH1 as novel component of the NOD1-pathway. (A) Combined Z-scores of the primary-hits in the counter screen with TriDAP (NOD1,ordinate) and TNF (abscissa) activation. (B) Flow-chart representing the screening procedure. Number of hits (genes) of each step is indicated. (C) Final hitlist ranked according to the THP1 results (best). Candidates validated in all steps and not affecting TNF signaling are shown in bold. Z-score were normalized to control siRNAs set to 0. (see also Figures S1, S2 and Table S1).

A druggable-genome siRNA-library (a sub-library of the human genome covering approximately 7000 genes with known protein domains) containing four independent siRNAs per gene was screened in quadruplicate for hits inhibiting NOD1-mediated NF-κB activation upon treatment with the NOD1-specific elicitor TriDAP (Figure S1A). After quality control and elimination of toxic siRNAs, preliminary candidates were selected using a probability-based algorithm (redundant siRNA activity; RSA) [17] (Table S1). Statistical analyses confirmed the high reproducibility of the results and the robustness of the assay controls (p65 and the non-targeting Allstars siRNA) (Figure S2A). The top 435 of the RSA-ranked candidates with at least two hit siRNAs were differentially tested for TriDAP- as well as TNF-induced NF-κB activation (referred to here as “validation screen” and “counter screen”, respectively), using two independent siRNAs in HEK293T cells (Figure 1A and S1B). Knock-down of 173 genes for this set displayed an inhibitory effect on TriDAP-induced NF-κB activation. Of those, 66 genes were specifically involved in NOD1 signaling in HEK293T cells, i.e. they did not significantly affect TNF-mediated NF-κB activation (Figure 1B, Table S1). Among them, 28 known regulators of NF-κB, partly with known specificity for NOD1 signaling, could be retrieved, confirming the validity of the screening results (Table S1). Gene ontology (GO) enrichment analysis revealed that GO terms linked to immune function, and in particular to NLR function, were significantly overrepresented among the preliminary hits (Figure S2B). In-depth analysis using Ingenuity pathway analysis highlighted that 56 of the 435 preliminary screen hits (12.9%) are known components of NF-κB signaling (Figure S2C). Among these 56 preliminary hits, 28 could be validated in the HEK293T validation screen, of which 14 were not influencing TNF-α-induced NF-κB activation.

In order to further minimize false positive hits due to off-target effects of the used siRNAs and also to exclude a cell type specific bias, the 435 preliminary candidates were further tested for their effect on endogenous NOD1-mediated NF-κB activation in human myeloid THP1 cells (THP1-blue reporter line) (Figure S2D), revealing a cluster of genes showing functional interactions as revealed by STRING analysis (Figure S2E). The results confirmed 28 genes that showed an effect on NOD1-mediated NF-κB activation in both HEK293T and THP1 cells. Among those, receptor-interacting serine/threonine-protein kinase 2 (RIPK2), NOD1, transcription factor p65 (RELA), X-linked inhibitor of apoptosis protein (XIAP), deltex 4 (DTX4), calreticulin (CALR), olfactory receptor family 12, subfamily D, member 2 (OR12D2), and ring finger protein 31 (RNF31) were the strongest candidates (>3 S.D.). Exclusion of the genes that affected TNF-induced NF-κB activation in HEK293T cells resulted in a short list of 18 genes (Figure 1C, Table S1). Although this candidate-list obtained in THP1 cells differed from that derived from HEK293T cells, the genes RIPK2, XIAP, the uracil nucleotide/cysteinyl leukotriene receptor (GPR17), SSH1, the snail family zinc finger 1 (SNAI1), and CHUK (IKKα) were confirmed as hits in both cell lines by this highly stringent procedure (Figure 1C,Table S1). Identification of RIPK2, XIAP and the inhibitor of nuclear factor kappa-B kinase subunit alpha (IKKα), all of which have recently been linked to the NOD1 pathway [7], [18], [19] validated the success of the screening procedure.

To demonstrate that we can reversely reproduce the findings from the screen, XIAP was silenced with a screen-independent siRNA. This strongly impaired NF-κB activation upon both NOD1 or NOD2 stimulation in HEK293T cells (Figure S3A and B) and THP1-blue cells (Figure S3C). Furthermore, TriDAP- or MDP-induced IL-8 secretion in THP1 cells was significantly reduced (Figure S3D). Taken together, our screen validated XIAP as an essential component of the NOD1 signaling pathway, in line with previous reports showing an involvement of XIAP in NOD1 and NOD2 signaling [7], [18]. XIAP was shown to mediate linear ubiquitylation of RIP2 [18] mediated by the so-called LUBAC complex. Of note, our screen also highlighted one component of the LUBAC complex, RNF31 (HOIP) as a strong candidate (Table S1), which was independently identified in a recent screen for components of the NOD2 signaling pathway [20]. Notably, we identified three novel genes, namely the uracil nucleotide/cysteinyl leukotriene receptor GPR17, the cofilin phosphatase SSH1 and the transcriptional repressor SNAI1 that hitherto had not been reported in the context of NOD1 signaling.

### SSH1 specifically contributes to NOD1- and NOD2-mediated inflammatory responses

**Figure 2:**
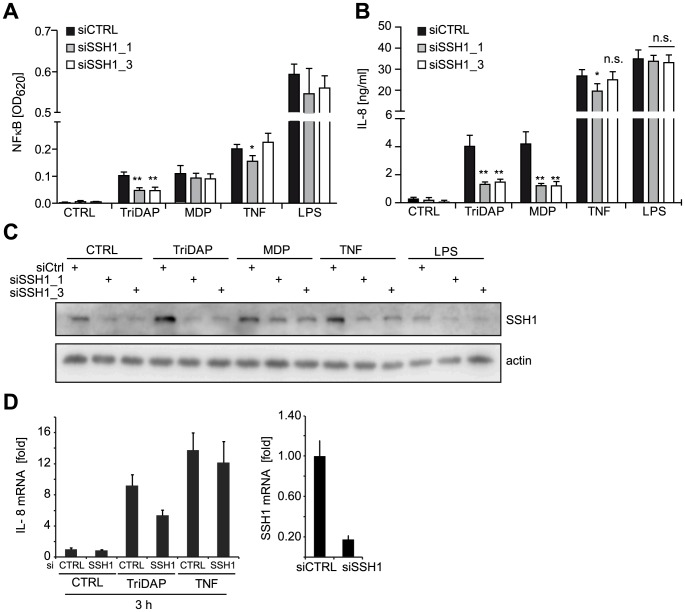
SSH1 is essential and specific for NOD1-mediated signaling. (A–B) PMA differentiated THP1-blue cells were treated for 72 h with a non-targeting (siCTRL) or two SSH1-specific siRNA duplex and incubated with TriDAP (10 µg/ml), MDP (10 µg/ml), TNF (0.1 µg/ml), or LPS (0.05 µg/ml). (A) NF-κB activation was measured by SEAP secretion. (B) IL-8 levels in the culture supernatants of the cells from (A). Values are mean + S.D. from two independent experiments conducted in triplicates. Significance was calculated by student's t-test (unpaired, two-tailed) *p<0.05, **p<0.005. n.s.: not significant. (C) Immunoblot of one of the experiments from (A), probing for SSH1 and actin as loading control is shown. (D) Early effects of the knock-down of SSH1. IL-8 (left panel) and SSH1 (right panel) mRNA levels in THP1-blue cells treated as inducted were measured by qPCR. Mean + S.D. from triplicate measurements of one representative experiment is shown (see also Figure S4).

Among these stringently validated hits, the phosphatase SSH1 (Table S1), a key regulator of actin dynamics (reviewed in [21]) caught our attention. In order to validate SSH1 as a critical component of NOD1 signaling, we depleted the protein by two different siRNAs in myeloid-like differentiated THP1-blue cells and measured NF-κB activity and IL-8 release upon treatment with the NOD1, NOD2, TLR4, and TNFR agonists TriDAP, MDP, LPS, and TNF, respectively (Figure 2A and B). This revealed a significant reduction of NOD1- and NOD2-mediated inflammatory responses (Figure 2A and B) concurrent with a reduction in SSH1 levels by siRNA treatment (Figure 2C). In contrast, TNF- and TLR4-induced responses were not highly significantly affected by reduced SSH1 levels (Figure 2A and B). Similar results were obtained in THP1 cells not containing the reporter construct, showing that NOD1-mediated release of several key inflammatory cytokines was reduced upon SSH1 depletion (Figure S4).

Immunoblotting showed that both siRNAs decreased the expression of SSH1 and revealed that SSH1 protein levels were increased upon PAMP stimulation (Figure 2C). To evaluate the effect of SSH1 knock-down at early time points after activation, we measured IL-8 and SSH1 mRNA levels 3 h after TriDAP or TNF stimulation in THP1-blue cells. This showed that the reduction in SSH1 correlated with reduced IL-8 mRNA levels when cells were activated by TriDAP but not when stimulated with TNF (Figure 2D).

**Figure 3:**
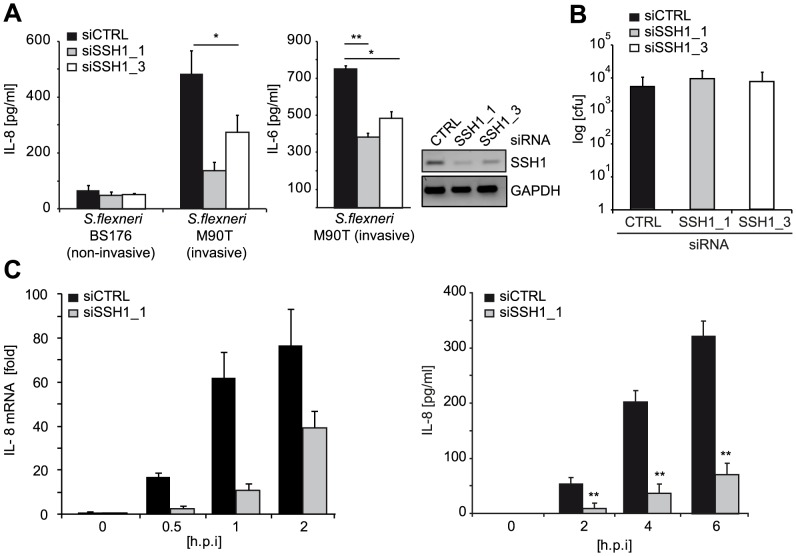
SSH1 is involved in early *Shigella*-induced pro-inflammatory responses. (A) HeLa cells were transfected with CTRL or SSH1 siRNAs and incubated for 72 h and infected with *Shigella flexneri* M90T or BS176 (MOI = 10). IL-8 and IL-6 (right panel) secretion was determined 6 h post infection by ELISA. Values are mean + S.D. (n = 2), representative of three independent experiments. Knock-down efficiency was determined by RT-PCR (right panel). (B) Intracellular bacteria were counted after gentamycin treatment of the cells treated as in (A) 2 h post infection. (C) HeLa cells treated for 72 h with siCTRL (filled bars) or siSSH1_1 (gray bars) were analyzed at the indicated time-points after infection with *S. flexneri* M90T afaE. Quantitative PCR of cDNA normalized to GAPDH expression and IL-8 concentration in the supernatant (right panel), mean + S.D. (n = 3) are shown. Significance was calculated by student's t-test (unpaired, two-tailed) *p<0.05, **p<0.005 (see also Figure S5).

To elucidate the effect of SSH1 on physiological activation of NOD1 by bacteria, we infected HeLa cells with the Gram-negative invasive bacterial pathogen *Shigella flexneri*, which is sensed primarily by NOD1 in these cells. Depletion of SSH1 mRNA by the two SSH1-specific siRNA duplexes resulted in significantly impaired IL-8 and IL-6 production 6 h post infection with the invasive *S. flexneri* strain M90T (Figure 3A). Notably, this was not due to reduced bacterial invasion and replication, as demonstrated by gentamicin protection assays (Figure 3B). To decipher if SSH1 is involved preferentially in early or late events during the inflammatory response to *S. flexneri*, we measured *IL-8* mRNA and IL-8 release at different time points after bacterial infection. Depletion of SSH1 (Figure S5A) led to greatly reduced IL-8 transcription as early as 30 min after infection (Figure 3C). Two hours post infection, when IL-8 became measurable in the supernatant of infected cells, a significant difference in IL-8 secretion was observed between cells treated with SSH1 targeting siRNA and those treated with a non-targeting control siRNA (Figure 3C). This suggests that SSH1 is already involved at early times of NOD1 activation. As expected, the knock-down of SSH1 led to a strong increase in phosphorylation of its substrate cofilin at serine 3 (Figure S5B), proving that SSH1 is active in our cellular system. To rule out that the effects described above were related to the malignant origin of the cell lines, we assessed the phenotype of SSH1 depletion in primary human dermal fibroblasts. In line with the results obtained with cell lines, in primary human dermal fibroblasts NOD1-mediated IL-8 secretion was also significantly reduced upon SSH1 knock-down, whereas SSH1 depletion affected TNF-induced IL-8 secretion to a lesser extent (Figure S5C).

Taken together, our data identified SSH1 as an essential component of NOD1-mediated activation of pro-inflammatory responses in human myeloid and epithelial cells.

### SSH1 interacts with NOD1 at F-actin structures

**Figure 4:**
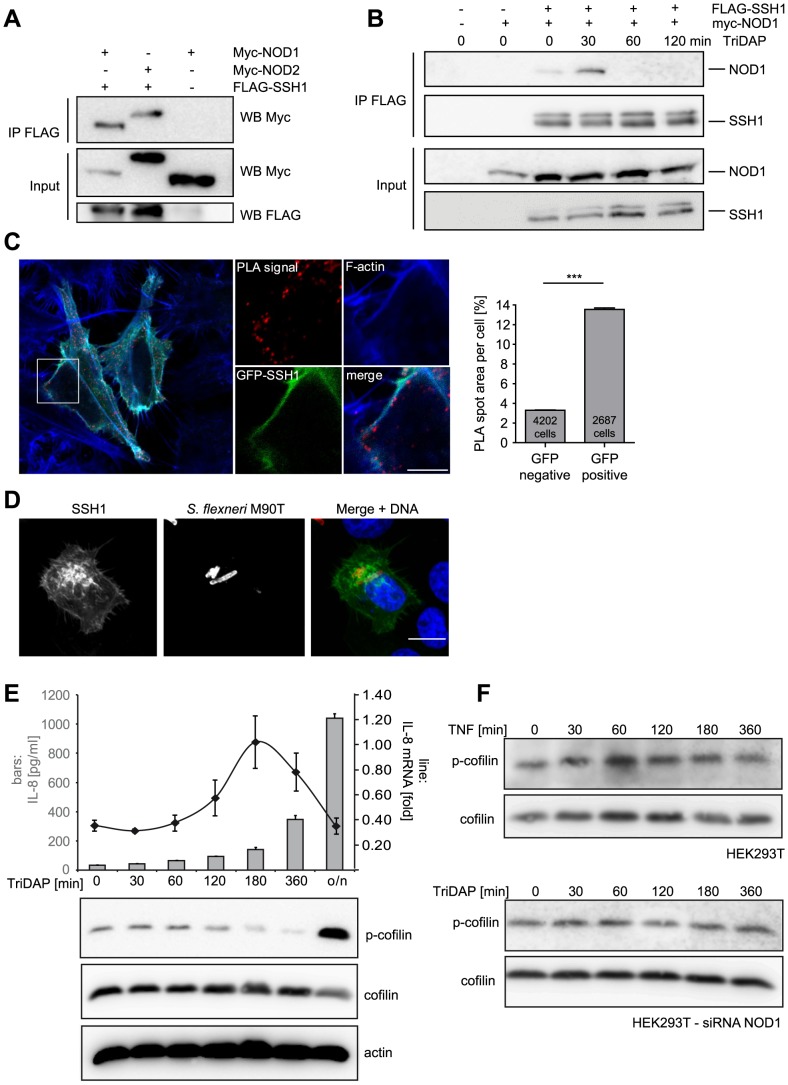
SSH1 interacts with NOD1 and regulates cofilin phosphorylation after NOD1 activation. (A) Co-immunoprecipitation of ectopically expressed SSH1 with NOD1 and NOD2 in human HEK293T cells. Flag-SSH1 was precipitated using a Flag-specific antibody in all samples. (B) Co-immunoprecipitation kinetic of NOD1 and SSH1 in HEK293T cells treated for the indicated time with 500 nM TriDAP. Flag-SSH1 was precipitated using a Flag-specific antibody. (C) *In situ* PLA detection of the interaction between GFP-SSH1 (shown in green) and Flag-NOD1 in transiently transfected HeLa cells. Protein–protein interactions are visualized as small, distinct red spots (PLA signals). F-Actin (blue) was stained with Alexa633-labeled phalloidin. Shown is a single z section. Scale bar 5 µm. Right panel: Quantification of the total spot area per cell. Significance was calculated by student's t-test (unpaired, two-tailed), p<0.0001. (D) Indirect immunofluorescence study of ectopically expressed myc-SSH1 in HeLa cells after 30 min of infection with *S. flexneri* (MOI = 10). Bacteria were stained with a LPS specific antibody and are shown in red in the merge image together with DNA staining in blue. Bar: 10 µm. (E) HEK293T cells expressing low amounts of NOD1 were stimulated with TriDAP. IL-8 release (bars) was measured by ELISA (accumulation in supernatant since t = 0) and IL-8 mRNA (line) by qPCR (upper panel). Values are mean + S.D. (n = 3). Cofilin phosphorylation (S3) was monitored by Western Blot analysis (lower panels). Probing for total cofilin and actin served as loading controls. One representative experiment out of three is shown. (F) p-cofilin and total cofilin protein levels were analyzed in HEK293T cells at the indicated time-points after stimulation with 50 ng/ml TNF (upper panel). Alternatively NOD1 was silenced by siRNA treatment for 48 h and cells were stimulated with TriDAP (lower panels) (see also Figure S6).

SSH1 was reported to bind F-actin and to co-localize with actin stress fibres [22], [23]. We confirmed that ectopically expressed SSH1 partly co-localized with F-actin in HeLa cells that stably express YFP-NOD1. Although the two proteins showed slightly different localization patterns, a partial co-localization of SSH1 and NOD1 was also observed in confocal imaging (Figure S6A). In co-immunoprecipitation experiments of transiently expressed SSH1 and NOD1/2 proteins, both NOD1 and NOD2 co-precipitated with SSH1 (Figure 4A), validating that both NLR proteins are able to form a complex with SSH1. Upon activation of NOD1 by TriDAP we observed a change in the stoichiometry of this complex with an increase in affinity about 30 min after TriDAP stimulation and reduced binding at later time points (Figure 4B). To analyse this protein-protein interaction in more detail we employed *in situ* proximity ligation assays (PLA). We found that GFP-SSH1 and Flag-NOD1 associated predominantly at F-actin positive structures (Figure 4C). Quantitative analysis using high throughput microscopy revealed that the area covered by PLA spots was approximately 4-fold higher in cells expressing GFP-SSH1 and Flag-NOD1 than in neighbouring GFP-negative cells, confirming the specificity of the signal (Figures 4C and S6B). Moreover, the phosphatase-dead mutant C393S of SSH1 gave similar results in the PLA assays as WT SSH1 (Figure S6C) showing that association of SSH1 and NOD1 was not dependent on the phosphatase activity of SSH1. Accordingly, SSH1 was not involved in membrane targeting of NOD1 and NOD2 in HeLa cells, as the sub-cellular localization of transiently transfected NOD1 and NOD2 were not markedly changed in cells with highly reduced SSH1 expression compared to controls (Figure S7A and S7B).

It was reported that in epithelial cells SSH1 is enriched at the entry foci of *Salmonella* [24]. Likewise, NOD1 is enriched at entry sites of *S. flexneri* [9]. We thus analyzed the localization of SSH1 in *S. flexneri*-infected cells, revealing an enrichment of SSH1 at the bacterial entry foci (Figure 4D).

We previously reported that depolymerization of F-actin by the mycotoxin cytochalasin D enhances NOD1-mediated NF-κB activation [9]. Based on our results, we hypothesized that the impact of SSH1 on NOD1 signaling might be mediated by cofilin-controlled actin remodeling and that the formation of a SSH1-NOD1/2 complex might control the local NOD1/2 activity at sites of bacterial entry. To address this, we monitored SSH1 activity indirectly by measuring phosphorylation of its substrate cofilin at serine 3 (p-cofilin) after TriDAP-induced activation of NOD1. TriDAP treatment of HEK293T cells expressing low levels of NOD1 strongly induced IL-8 release from these cells (Figure 4E). Interestingly, basal p-cofilin levels decreased at the time when *IL-8* transcription was highest (about 180 min post TriDAP treatment) (Figure 4E). After longer periods of incubation (16 h), p-cofilin levels increased above the level seen in untreated cells (Figure 4E). The slower kinetics in these experiments compared to *S. flexneri* infection in HeLa cells (Figure 3) is likely due to the different kinetics of TriDAP uptake in HEK293T cells [5]. Notably, no obvious change in p-cofilin levels was observed after stimulation of HEK293T cells with TNF, although TNF induced high IL-8 release (Figure 4F and S6D). Moreover, in cells in which NOD1 was depleted by siRNA TriDAP treatment did not robustly influence p-cofilin levels over time (Figure 4F). Accordingly, TriDAP failed to induce IL-8 secretion form these cells (Figure S6D). Moreover, expression of SSH1 did not result in increased basal NF-κB activity (Figure S8B).

Collectively, these data provide evidence that NOD1 signaling relies on the presence of SSH1 that acts downstream of NOD1 and involves the activation of cofilin.

### NOD1 signaling is connected to changes in the actin cytoskeleton

**Figure 5:**
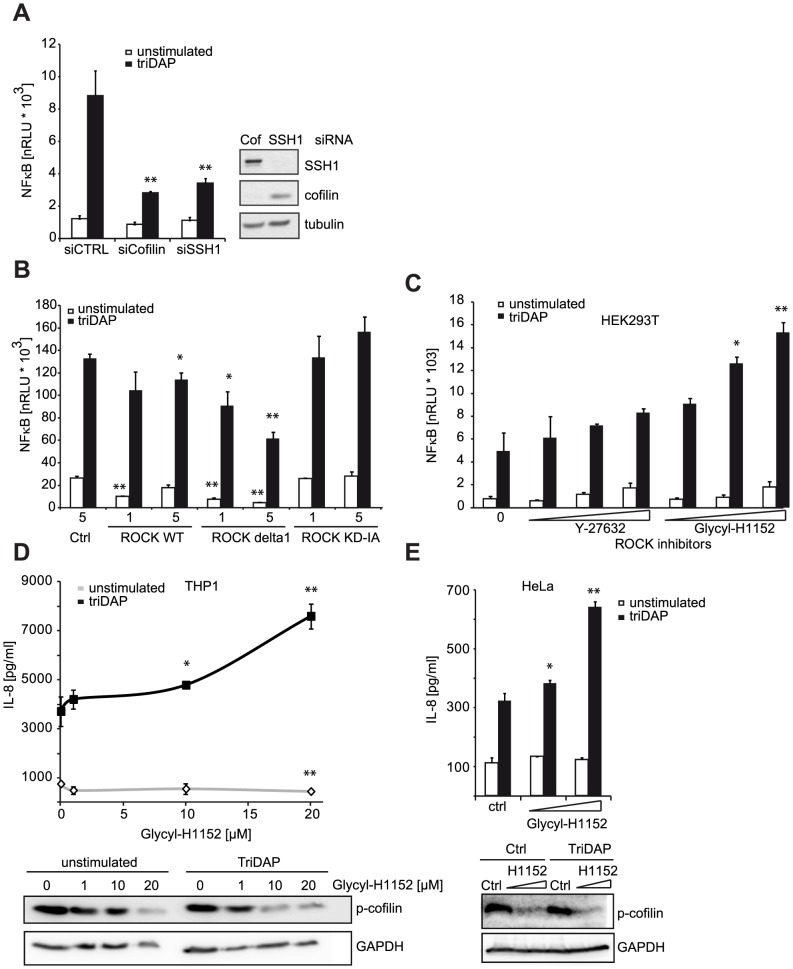
ROCK activity influences NOD1 signaling. (A) HEK293T cells were transfected with CTRL or siRNA specific for the indicated target and incubated for 48 h. Subsequently, cells were transfected with the NF-κB luciferase reporter and NOD1 and stimulated with TriDAP (filled bars). After 16 h, cells were lysed, luciferase activation was determined and normalized with the β-galactosidase values (nRLU). Values are mean + S.D. (n = 3). Knock-down of the respective target is shown by immunoblot in the right panel. (B) Effect of the expression of constitutively active (CA) and dominant negative (DN) forms of ROCK1 on NOD1-mediated NF-κB activity measured by luciferase reporter assays in HEK293T cells. Values are mean + S.D. (n = 3). (C) NOD1-induced NF-κB activity measured by luciferase reporter assays in HEK293T cells for 6 h with the ROCK inhibitors Y-27632 and Glycyl-H1152. Values are mean + S.D. (n = 3). (D) THP1 cells were treated with the indicated amount of ROCK inhibitor Glycyl-H1152 for 6 h and TriDAP-induced IL-8 secretion was determined by ELISA. Mean + S.D. (n = 3) is shown. Cells were lysed and p-cofilin was detected by Western Blot analysis using a specific antibody (lower panel). Detection of GAPDH served as loading control. (E) Same experimental setting as (D), using HeLa cells stimulated with 10 µg/ml TriDAP. Mean + S.D. is shown. * p<0.05, ** p>0.005 compared to Ctrl (see also Figure S8).

We next asked if other components of the cofilin regulatory network also contributed to NOD1 signaling outcome. Using siRNA-mediated knock-down we confirmed that reduction of cofilin resulted in a similar perturbation of NOD1 signaling as SSH1 knock-down (Figure 5A). Cofilin is regulated primarily by phosphorylation through the kinases LIMK1/2, which is counteracted by the phosphatase activity of SSH1. Both SSH1 and LIMK1/2 are themselves regulated by phosphorylation events mediated by protein kinase D (PKD), ROCK1/2 and PAK1/4, respectively (reviewed in [25]). Expression of a dominant negative form of ROCK1 (KD-IA, which lacks kinase and Rho binding activity) enhanced, albeit not significantly, NOD1-mediated responses in a dose-dependent manner, whereas a constitutively active mutant of ROCK1 (delta1) significantly inhibited signaling by NOD1 (Figure 5B). To further substantiate this, we tested the effect of two potent chemical inhibitors of ROCK - Y-27632 and Glycyl-H1152 - on NOD1-mediated signaling. In HEK293T cells, both inhibitors enhanced TriDAP-induced NOD1-mediated NF-κB activation in a dose dependent manner, this enhancement was significant in the case of Glycyl-H1152 (Figure 5C). By contrast, both compounds led to a significant reduction of TNF-induced NF-κB responses (Figure S8A). Chemical inhibition of ROCK also led to higher NOD1-mediated pro-inflammatory responses in TriDAP stimulated HeLa and THP1 cells (Figures 5D and E). ROCK inhibition correlated in a dose dependent manner with reduced levels of p-cofilin, suggesting that increased cofilin activity causes this effect on NOD1 signaling. As shown before, stimulation of cells with TriDAP further enhanced dephosphorylation of cofilin (Figures 5D and E). To provide direct evidence that the phosphatase activity of SSH1 is responsible for modulation of NOD1 activity, we overexpressed the phosphatase-dead mutant C535S of SSH1 in HEK293T cells. In line with our hypothesis, this did not affect NOD1-mediated signaling (Figure S8C).

**Figure 6:**
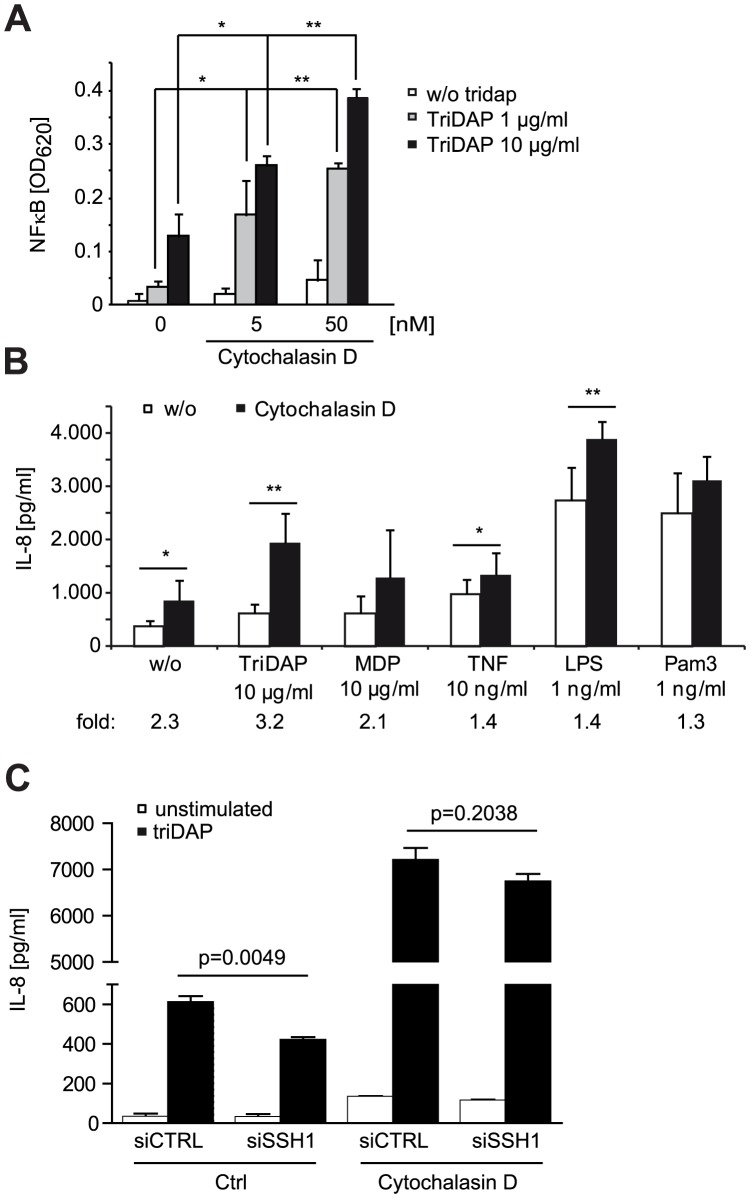
NOD1 signaling is dependent on F-actin. (A) Effect of 6 h cytochalasin D (cytoD) treatment on TriDAP/NOD1 induced NF-κB activation in PMA differentiated THP1-blue reporter cells. Mean + S.D. (n = 3). Significance was calculated by student's t-test (unpaired, two-tailed) *p<0.05, **p<0.005 (compared to 0 nM cytoD). (B) IL-8 secretion of THP1 cells treated for 6 h with the indicated PAMP in the presence or absence of cytochalasin D. Shown is the mean of triplicates from two independent experiments + S.D. (n = 6). The fold induction of IL-8 by cytoD is depicted below. (C) SSH1 knock-down effect on cytoD enhanced NOD1 signaling. Differentiated THP1 cells were treated with cytoD for 6 h and stimulated with 10 µg/ml TriDAP (black bars) or mock treated (open bars). IL-8 secretion measured in the supernatant after 6 h of incubation is shown, mean + S.D. (n = 3), * p<0.05, ** p<0.005.

These results show that perturbation of the cofilin pathway at different levels affected NOD1 signaling, suggesting that NOD1 signaling relies on cofilin-mediated changes in actin remodeling. Accordingly, NOD1 signaling induced by actin polymerization-perturbing mycotoxins should be SSH1 independent. As reported for HEK293T cells [9], we found that depolymerization of F-actin using cytochalasin D strongly enhanced NOD1-mediated signaling in THP1 cells (Figure 6A). In line with previous reports, cytochalasin D enhanced IL-8 release induced by other PAMPs about 2-fold [26], [27]. However, in the case of NOD1 activation by TriDAP, a significantly higher increase to ∼3-fold was observed (Figure 6B). Notably, this was not the case upon activation of NOD2 by MDP. Next, we depleted SSH1 expression in THP1-blue cells and subsequently disturbed actin polymerization by cytochalasin D treatment. Knockdown of SSH1 significantly reduced NOD1-mediated NF-κB activity in cells treated with TriDAP, however, treatment with cytochalasin D rescued the effect of SSH1 depletion on TriDAP-induced NOD1 activation (Figure 6C). This strongly suggests that F-actin affects NOD1 signaling downstream of SSH1.

Taken together, our data support that NOD1 activation and induction of pro-inflammatory responses requires actin remodeling controlled by the SSH1 and cofilin network.

## Discussion

Using an unbiased high-throughput siRNA screen, we identified novel factors involved in the regulation of the NOD1 signaling cascade. The validity and quality of our screening approach is highlighted by the fact that the screen identified many factors involved in canonical NF-κB and/or NOD1 signaling. Most prominently, the primary screen validated the proteins RIPK2 (reviewed in [2]), IKKα [19], IKKβ (reviewed in [2]), TAB2 [28], [29], RNF31 [30], p50 and RELA [31] as essential positive regulators of NOD1 signaling. Beside RIP2, XIAP ranked the highest throughout the whole screening procedure among the NOD1 specific hits. In line with a recent publication, we observed blunted responses of XIAP depleted cells to stimulation with NOD1 and NOD2 elicitors [7]. A recent study now provides the framework for the function of XIAP in this process, by showing that it acts as a ubiquitin ligase for RIP2, catalyzing linear ubiquitylation events, at least in NOD2 signaling [18]. By using *S. flexneri* as an infection model we could recently demonstrate the physiologic relevance of these findings *in vitro* and *in vivo*, underscoring the impact of XIAP on anti-bacterial immunity [32].

With high confidence the screen identified a central regulator of actin cytoskeletal dynamics, the phosphatase SSH1, as novel component of the NOD1 signaling cascade. Notably, SSH1 was recently also identified as a potential hit in two independent siRNA screening efforts searching for NOD1 and NOD2 signaling components, although it was not validated in neither of these studies [20], [33]. SSH1's best described function is the dephosphorylation and subsequent activation of the actin depolymerization factor cofilin. Its activity is known to be counter-acted by LIMK1 and LIMK2, which phosphorylate and thus inactivate cofilin on serine 3 (reviewed in [21]). Using a highly stringent and unbiased multilayer screening approach offers high confidence in the obtained data. However, candidates can be overlooked due to loss because of mismatch of quality criteria. This likely explains why cofilin, ROCK1, ROCK2 and others, although being represented in the screened library, were not identified as validated hits.

We confirmed the function of SSH1 in NOD1 signaling by independent siRNA knock-down experiments in different human cell lines and primary human dermal fibroblasts. This validated that silencing of SSH1 significantly impaired NOD1-mediated responses in human cells triggered by TriDAP and infection with the invasive bacterial pathogen *S. flexneri*. Consistent with the screen data, SSH1 knock-down affected TNF and LPS-induced NF-κB activation to a far lesser extent than NOD1- and NOD2-mediated responses, showing that SSH1 contributes to NOD1 and NOD2 signaling in a rather specific manner. SSH1 thus contributed to NOD1 signaling at early times. Taken together, our results suggest that SSH1 affects NOD1 signaling through its phosphatase activity. This is best evidenced by the observation that NOD1-induced activation of *IL-8* transcription was accompanied by a reduction of cofilin phosphorylation and the lack of effect of a phosphatase-dead mutant of SSH1 on NOD1 signaling. Our data do not allow drawing conclusions on how SSH1 activity is triggered in this process. However, the observed complex formation of SSH1 and NOD1, that exhibited changed stochiometry upon triggering of NOD1 by its elicitor TriDAP, makes it tempting to speculate that binding of NOD1 to a SSH1-containing complex initiates local SSH1 activation. Noteworthy, we observed that treatment with several PAMPs resulted in enhanced SSH1 proteins levels in THP1 cells. A plausible interpretation of this finding might be that higher SSH1 levels might render host cells more prone for enhanced and more rapid NOD1-mediated immune signaling. Further research will help to address this and to establish the biological significance of this finding.

Invasive bacteria, such as *Shigella* and *Salmonella*, depend on a tightly controlled spatial and local reorganization of F-actin at the plasma membrane to gain entry into the host cell. NOD1 is well-recognized as an important sensor of bacterial invasion and we showed earlier that it co-localizes with F-actin at the cell membrane and that depolymerization of F-actin by cytochalasin D augments NOD1 signaling in epithelial cells [9]. In the cell, actin dynamics are controlled by a balance between the activities of the small GTPases RhoA and Rac1 and it has been reported that changing their activity affects NOD1 and NOD2 signaling [12], [13], [34]. The pathogenic bacterium *Klebsiella pneumonia* seems to inhibit Rac1 activity to trigger NOD1 signaling, resulting in a dampened innate immunity response [34]. Additionally, the Rho activator guanine nucleotide exchange factor H1 (GEF-H1) was identified as an essential component of NOD1-mediated signaling in response to *Shigella* and muropeptides [11]. In all these studies, the mechanistic link to the modulation of NOD1 signaling, however, was not conclusively identified. Our results show that NOD1 signaling competence relies on actin remodeling via cofilin. Activation of NOD1 by chemical ligands reduced cellular p-cofilin at the time when pro-inflammatory signaling was induced. Because SSH1 and NOD1 directly interact at the plasma membrane, SSH1 might act as a local platform to recruit NOD1 to the entry site of pathogens. The fact that cofilin activity is also modulated by RhoA and Rac1 activity [25] strongly suggests that SSH1 and cofilin are key effectors that link NOD1 activation to perturbations in the network of actin regulation. In support of this notion, our data show that “sterile” interference with the cofilin pathway at several levels, as well as pharmacological disruption of the actin cytoskeleton, modulated NOD1 signaling outcome. For example, we observed enhancement of NOD1 signaling upon overexpression of a dominant negative protein of the RhoA effector kinase ROCK or pharmacological inhibition of ROCK kinases. We cannot formally exclude that the inhibitors affected other cellular targets. However, in conclusion all data strongly support that interfering with the F-actin network downstream of RhoA and upstream of cofilin profoundly alters NOD1 signaling. Finally, we observed that induction of NOD1-mediated responses by depolymerization of F-actin is independent of SSH1. Taken together, these experiments showed that NOD1 signaling outcome correlated directly with cofilin activity.

F-actin depolymerization by cytochalasin D in myeloid cells was shown to affect NF-κB signaling in a broader manner, as confirmed by our results [26], [27]. It should be noted, that a comparative analysis including multiple PRRs was not conducted in these studies. We observed a much higher synergy on the NOD1-induced NF-κB activation, indicating that NOD1 signaling is particularly prone to changes in actin dynamics. Surprisingly, also NOD2-induced IL-8 responses were less strongly enhanced by cytochalasin D in myeloid cells compared to NOD1, although SSH1 interacted with NOD2 and knock-down of SSH1 also affected NOD2-mediated signaling. Further research is needed to define the surprising differences in the contribution of the actin cytoskeleton and SSH1 to NOD1 versus NOD2 signaling. Regulation of PRR signaling by actin is not without precedence, as a role for Rac1 in regulation TLR2 function has been shown before [35]. Furthermore, there are interesting parallels in the regulation of mammalian and plant NLRs, suggesting that effector triggered immunity (ETI) in plants brought about by activation of plant NLR proteins, is also intimately linked to actin dynamics. In *Arabidopsis* there is genetic evidence that the actin remodeling protein ADF-4 negatively affects RPS4-mediated ETI responses, although the authors do not disclose if this is linked to changed actin dynamics [36].

Very recently it has been proposed that NOD1 acts as a sensor of Rac1 and CDC42 activity induced by bacterial type III effector proteins, such as the Salmonella virulence factor SopE [10]. It is, however, still elusive how this is mechanistically linked to changes in NOD1 activity. In any case, bacterial- induced perturbation of actin dynamics that are needed for bacterial cell entry, in particular in epithelial cells, would result in enhanced NOD1-mediated inflammatory responses. The data reported here provide novel insights into the underlying mechanisms showing that SSH1-mediated actin remodeling is a central component of NOD1 activation and innate immune responses.

## Materials and Methods

### Cells and bacteria

HEK293T, HeLa, THP1 and THP1-blue (InvivoGen, France) cells were cultured as described in in [37]. For immunofluorescence, a HeLa line stably expressing EGFP-tagged NOD1 was generated. All cell lines were continuously tested for absence of mycoplasma contamination by PCR. Primary human dermal fibroblasts were obtained as previously described [38].

### Plasmids and reagents

Plasmids encoding myc tagged human NOD1 and NOD1 were generated by PCR cloning in a pCDNA3.1 backbone.

SSH1 encoding plasmids are described in [39]. Plasmids encoding ROCK and mutants are described in [40] and RhoA and Rac1 plasmids were a kind gift from Monilola Olayioye (University of Stuttgart). ROCK inhibitors and cytochalasin D were purchased from Tocris.

### siRNA treatment

siRNA-based knock-down in HeLa and THP1 cells was performed as described previously in [37]. siRNAs used: SSH1_1 : SI00123585, SSH1_3: SI00123599 (Qiagen) and AllStars negative control (Qiagen).

### Bacterial infection with *Shigella flexneri*

For the infection with *S. flexneri*, HeLa cells were seeded in 24-well plates. Infection was performed using the strain M90T afaE as described previously [41]. Gentamycin (100 µg/ml) was added to the cells 30 min after addition of the bacteria. As control, a non-invasive derivative (BS176 afaE) was used.

Uptake of *S. flexneri* was analyzed by lysis of infected cells in 0.5% SDS/H_2_O. Serial dilutions of cell lysates were plated onto trypticase soy broth bacto agar plates without antibiotics and incubated at 37°C for 24 h. Colonies were counted and the recovery was determined.

### Co-immunoprecipiation and immunoblot analysis

Immunoprecipitations and immunoblots were conducted using 9E10-agarose (Santa Cruz) essentially as described previously [41]. Cells were transiently transfected with SSH1 constructs and NOD1 or NOD2 expression plasmids for 24 h. Antibodies used: mouse anti-Flag (Sigma, M2), mouse anti-myc (Santa Cruz, 9E10), HRP-conjugated goat anti-mouse IgG (Bio-Rad) and HRP-conjugated goat anti-rabbit IgG (Bio-Rad), rabbit anti-SSH1 (Abcam, 76943), rabbit anti-cofilin P-S3 (Cell Signaling, 3313), rabbit anti-cofilin (Cell Signaling, 5175), rabbit beta-actin HRP-conjugated (Santa Cruz, sc-47778 HRP), rabbit anti-alpha tubulin (Sigma, T7816), rabbit anti-GAPDH (Santa Cruz, 25778).

### Luciferase reporter assays

Activation of inflammatory pathways was measured using a luciferase reporter assay described previously [16]. The means and standard deviations were calculated from triplicates.

### RNA preparation and RT-PCR

Total RNA was extracted from cells using the RNeasy kit (Qiagen). One µg of RNA was reverse transcribed using the First-Strand cDNA synthesis kit (Fermentas).

For quantitative PCR analyses, 50 ng cDNA was analyzed in a total volume of 25 µl using the iQ SYBR Green Supermix (Bio-Rad), according to the manufacturer's protocol. All quantitative PCR reactions were run on a Bio-Rad iQ5 cycler, and data were evaluated by the iQ5 system software (version 2.0) using the ΔΔCT method.

For quantification of SSH1, the following primers were used (5′-3′): CGTTGCGAAGACAGAATCAA and CTCCACAGTCGGAGAACCAT. Primer for IL-8, NOD1, NOD2, and GAPDH are described in [37].

### Indirect immunofluorescence microscopy

Cells were transfected using Lipofectamine 2000 (Invitrogen). After 16–24 h incubation, cells were fixed and processed as described previously [37].

DNA was stained using DAPI. Images were acquired on an Olympus Fluoview 1000 confocal microscope and processed using ImageJ.

### *In situ* proximity ligation assay

The proximity ligation assay was performed with the Duolink system (Olink bioscience). HeLa cells grown on collagen-coated coverslips were transfected and fixed 24 h post transfection in 4% paraformaldehyde for 15 min, washed, permeabilized with 0.1% Triton X-100, and blocked with blocking buffer (Olink bioscience) for 30 min at 37°C. The cells were incubated with the primary antibodies (rabbit GFP-specific antibody and mouse Flag-specific antibody) diluted in blocking buffer for 2 h. As a negative control, GFP-SSH1 and Flag-NOD1 transfected cells, which were incubated in antibody diluent without primary antibodies, were used. Incubation with secondary antibodies and ligation and amplification were done as recommended by the manufacturer. Cells were stained with HCS Cellmask deep red (Life Technologies) and mounted in mounting medium (Olink bioscience). PLA dots were acquired with a LSM710 confocal microscope (Zeiss). F-Actin labeling (Alexa633-coupled phalloidin, Life Technologies) was performed after PLA staining. Quantifications of PLA dots were performed with HCS/HTS – based automated PLA spot detection. In brief, images were acquired on a WiSCAN Hermes system (Idea Biomedical, Israel), equipped with a Olympus 20× 0.75 NA objective. The quantitative spot analysis was performed using the WiSOFT image analysis software (Idea Biomedical, Israel). Cells were segmented via the HCS cell mask deep red staining. After segmentation, cells were classified into GFP-positive- and negative cells using a threshold in the green channel. Spots were identified in the red channel and the total area covered by all spots in one cell was calculated. Spots outside of the cell mask were not considered.

### Detection of cytokines by ELISA

Measurement of cytokines was performed using the appropriate ELISA kits (Duoset, R&D) according to the manufacturer's instructions. Multiplex cytokine analysis was performed by flow cytometry using the human inflammation 2plex kit (eBioscience).

### Statistical analyses

Data were analyzed by two-sided Student-t test using Microsoft Excel 2007 and GraphPad Prism 5.04.

### Large scale siRNA NF-κB luciferase screen

The siRNA screen was performed using the human druggable-genome siRNA library from Qiagen (Hilden, Germany) consisting of four individual siRNAs for each gene. HEK293T cells were transfected with siRNA (20 nM) using HiPerFect (Qiagen) and treated with TriDAP (0.5 µM, InvivoGen, San Diego, CA, USA) or TNF (5 ng/ml), respectively, in case of the counterscreen.

For screening, cells with a passage number of 2 were used. The whole assay procedure was performed automatically using a Biomek FXP laboratory automation workstation (Beckman Coulter) in 384 well plates (Corning).

4 µl of 200 nM siRNAs (Qiagen) were pre-spotted on 384 well plates to allow reverse transfection of cells. All plates contained non-targeting (Allstars; Qiagen) as well as p65, NOD1, and PLK siRNAs as internal controls. For transfection, 8 µl medium per well were mixed with 0.25 µl HiPerFect and added to the siRNAs. The mixtures were incubated at RT for 15 min before adding 1,000 HEK293T cells in 30 µl medium.

After 48 h incubation the medium was changes and cells were transfected with the NF-κB-luciferase reporter system (11.6 ng β-gal plasmid, 7.03 µl NF-κB-luciferase plasmid, 0.135 ng NOD1-expression plasmid, 8.78 ng pcDNA-plasmid, and 0.0918 µl Fugene6 (Roche)). Subsequently, cells were stimulated with 0.5 µM TriDAP (InvivoGen) in a volume of 3 µl H_2_O. For the TNF-counterscreen, 5 ng/ml TNF were added instead. After stimulation, cells were incubated at 37°C and 5% CO_2_ for 16 h.

For read-out, cells were lysed by adding 30 µl 2xlysis buffer (50 mM Tris pH 8.0, 16 mM MgCl2, 2% Triton, 30% Glycerol, H_2_O) and subsequently mixed by pipetting. 35 µl of the lysate were then added to a white 384 well plate containing 35 µl reading-buffer (1× lysis buffer w/o Triton containing 0.77 µg/ml D-luciferin and 1.33 mM ATP). Subsequently, bioluminescence of the samples was measured with an Envision plate reader (PerkinElmer).

For β-gal read-out, 35 µl of ONPG-development buffer (4 mg/ml ONPG in 60 mM Na2HPO4, 40 mM NaH2PO4, 10 mM KCl, 1 mM MgSO4, pH 7.0) were added to the remaining lysate. After 15 min incubation at 37°C and 5% CO_2_, the absorbance of the samples at 405 nm was measured automatically with an Envision plate reader (PerkinElmer). Each individual siRNA was tested in 4 four biological replicates.

### THP1-blue siRNA screen

For screening, the siRNAs were pre-spotted on 384 well cell culture plates. All plates contained Allstars, p65, NOD1, and PLK siRNAs as internal controls. For transfection, 0.25 µl HiPerFect were added to the siRNAs. The mixtures were incubated at RT for 15 min, before THP1-blue cells and 0.1 µM PMA (Sigma, Munich, Germany) were added. The cells were incubated for 72 h, while the growth medium was exchanged twice a day.

Subsequently, cells were stimulated with TriDAP (10 µg/ml, InvivoGen). The cells were incubated for 16 h before read-out. For SEAP-detection, 10 µl supernatant per well were transferred to a plate containing 50 µl QUANTI-Blue SEAP detection medium (InvivoGen) and incubated for 5 h. 10 µl XTT reagent were added to the remaining 10 µl and incubated at 37°C for 1 h. Absorption at 632 nm and 485 nm respectively were measured using a PerkinElmer EnVision plate reader.

### Data analysis of siRNA screen

Data was processed using the CellHTS2 package [42], Bioconductor/R, and Excel (Microsoft). By dividing the luciferase signal (relative light units; RLU) by the β-gal signal (ABS405), normalized relative light units (RLU/ABS405 = nRLU) were achieved. To exclude experimental artifacts, all data from a given plate was excluded, if the average β-gal signal of the non-targeting controls (ABS405) was >2.5, <0.2, or had a standard deviation of >50%. Next, all wells showing a β-gal signal of <40% of the non-coding controls, supposedly due to low plasmid transfection efficiency or siRNA toxicity, were excluded from further analysis. Subsequently the nRLU where normalized relative to the inhibitory effect of the p65-control-siRNAs compared to the non-targeting controls (normalized percent inhibition; NPI) and median z-scores of the 4 biological replicates were calculated (centered to the median of non-targeting controls), using CellHTS2.

In the next step, the median z-scores of individual siRNAs were used to calculate a ranked gene list, using the redundant siRNA analysis algorithm; genes with less than two hit-siRNAs (‘OPI-hits’) were excluded (RSA) [17]. This list comprises genes leading to a decreased p65 activity, when knocked down (termed “inhibiting hits”).

For TNF-counter screening in HEK293T cells, as well as for hit validation in HEK293T and THP1 cells, the top 435 inhibiting hits were selected. For each of these genes, the two siRNAs showing the strongest effect in the screen were re-synthezised and assembled on 384-well plates (‘validation plates’).

To validate the results of the primary screen, the experiments were repeated as described above. Data analysis using CellHTS2 was done as described above; siRNAs were selected as ‘inhibiting Tri-DAP-hits’, if their median Z-score exceeded the median of non-targeting controls by more than two standard deviations.

To exclude unspecific hits, all siRNAs selected for validation were screened for their influence on TNF-induced p65 activation. Data analysis was done analogous to the Tri-DAP validation screen. ‘Inhibiting TNF-hits’ were excluded from further analysis.

Data from the THP1-blue screen consists of two parameters (QUANTI-Blue absorption at 632 nm for Tri-DAP response [QB], and XTT absorption at 485 nm for cell viability [XTT]) and was processed similar as described above: QB-signal of each well was normalized to cell viability (XTT), yielding nQB (normalized QUANTI-Blue absorption; QB/XTT = nQB). After quality control and outlier flagging, the three best experimental replicates were NPI-normalized to non-targeting and NOD1-control-siRNAs using CellHTS2, and median Z-scores were used for hit identification. All genes with two siRNAs showing a decrease of >1.5-fold standard deviation compared to the non-targeting control were regarded as validated inhibiting hits. A subset of these with one siRNA showing a decrease of >3.0-time standard deviation were categorized as ‘strong inhibiting hits’ (8 genes).

### Gene accession numbers

SSH1: Gene ID 54434; NOD1: Gene ID 10392; NOD2: Gene ID 64127; ROCK1: Gene ID 6093; ROCK2: Gene ID 9475.

## Supporting Information

Figure S1 **Flow-chart and graphical illustration of the basic screening protocol used in HEK293T cells.** (A) Layout of the screening procedure. (B) Schematic representation of the NOD1 signaling pathway. Related to Figure 1.
(PDF)

Figure S2 **Analysis of the screen data.** (A) Distribution of the non-targeting controls (green), positive control (p65, orange) and all tested siRNAs (blue). Left panel: Dot-plot. Right panel: Ranked presentation of all hits. (B) Gene ontology (GO) enrichment of the 435 preliminary hit genes retrieved by the primary screen compared to the druggable-genome library (background). Number of hit genes associated with enriched terms according to the GO of biological processes. Enrichment factors and negative log(P-values) are indicated in brackets. (C) Known regulators of NF-κB retrieved by the screens. Ingenuity Pathway analysis of 56 (out of 435) preliminary inhibiting screen hits already known to be involved in NF-κB regulation. Among them, 28 could be validated in the HEK validation screen (dark red: strong hit; light red: weaker hit; white: not validated). A sub group of 14 were not influencing TNF-α-induced NF-κB activation (blue borders). (D) Distribution of the siRNAs in the THP1-blue validation screen. Each dot represents the median Z-score of the experimental replicates after NPI-normalisation (Normalized Percent Inhibition) to the plate internal NOD1-controls. A positive value represents an inhibitory effect of the respective siRNA on NF-κB activation. THP1 hit-siRNAs are defined as exceeding 1.5 S.D. of CTRL from the median of the CTRL siRNAs. Z-scores were normalized to control siRNAs set to 0. CTRL: non-targeting controls. (E) STRING database analysis of the 28 hits from the THP1 screen. Genes, where the NPI values of both siRNAs exceeded 1.5 S.D. of CTRL from the median of CTRL were regarded as validated. Stronger associations are represented by thicker blue lines. Genes were clustered according to the Marcov cluster (MCL) algorithm. Related to Figure 1.
(PDF)

Figure S3 **Validation of XIAP as essential component of the NOD1 signaling pathway.** (A) HEK293T cells were transfected with CTRL or XIAP siRNA and incubated for 48 h. Subsequently, cells were transfected with the NF-κB luciferase RPS- containing NOD1 (left panel) or NOD2 (right panel) expression plasmids and stimulated with TriDAP (0.5 µM) or MDP (50 nM), respectively. After 16 h, cells were lysed, luciferase activation was determined and normalized with the β-galactosidase values (nRLU). Values are mean + S.D. (n = 3). (B) Confirmation of the XIAP knock-down on protein level. HEK293T cells were transfected with CTRL or XIAP siRNA. After 72 h, cells were lysed and XIAP protein levels were determined by Western blot using a XIAP-specific antibody. Detection of GAPDH served as loading control. (C) Differentiated THP1-blue cells were transfected with CTRL, NOD1, NOD2, or XIAP siRNA. Cells were incubated for 72 h and subsequently stimulated with TriDAP or MDP (10 µg/ml each), 16 h later, NF-κB activation was determined by measurement of SEAP activity in the supernatants. (D) In parallel, IL-8 secretion was determined by ELISA. Values are NF-κB activation [OD_620_] or IL-8 secretion [pg/ml], respectively, mean +SD (CTRL, XIAP: n = 3; NOD1, NOD2: n = 2). Data is representative for at least three independent experiments. Related to Figure 1.
(PDF)

Figure S4 **SSH1 knock-down effect in THP1 cells.** PMA differentiated THP1 cells were treated for 72 h with a non-targeting (siCTRL) or two SSH1 specific siRNA duplex. (A) IL-8 secretion was measured by ELISA after stimulation for 16 h with TriDAP (10 µg/ml), MDP (10 µg/ml), TNF (0.1 µg/ml), and or LPS (0.05 µg/ml) stimulation. (B) In parallel, SSH1 mRNA levels were determined by qPCR from the same cells. Values are Mean + S.D. (n = 3) representative of at least three independent experiments. * p<0.05. n.s.: not significant. (C) Cytokine release of 20 relevant inflammatory cytokines of the cells in (A) after TriDAP stimulation was analyzed by multiplex bead array. Changes in the cytokine response following SSH1 depletion by siRNA is shown for the proteins with the highest induction. Related to Figure 2.
(PDF)

Figure S5 **SSH1 knock-down phenotype in HeLa and primary human dermal fibroblasts.** (A) qPCR analysis of SSH1 levels in the experiment shown in Figure 3C. (B) Immunoblotting for cofilin S3-phosphorlyation in HEK293T cells after SSH1 knock-down. (C) Primary human dermal fibroblasts of early passages were transfected with the indicated siRNA for 72 h and subsequently treated with TNF, TriDAP, or infected with *S.flexneri* and IL-8 release was measured 6 h later by ELISA (upper panel). Percent IL-8 level compared to cells treated with the siCTRL SSH1 knock-down is shown by qPCR from the same cells (lower panel). Mean + S.D. (n = 3) representative of experiments from cells of two different donors is shown. Related to Figure 3.
(PDF)

Figure S6 **SSH1 co-localizes with Nod1.** (A) Indirect immunofluorescence images of HeLa cells expressing GFP-NOD1 and myc-SSH1. NOD1 signal is shown in green in the merge image together with the SSH1 signal in red and DNA staining in blue. (B) HCS/HTS – based PLA spot quantification. Images show cell segmentation with HCS cell mask stain (blue channel). Cell classification in EGFP-SSH1 positive/negative (green channel, indicated in blue channel) and automated PLA-spot detection (red channel). Scale bar 25 µm. (C) Quantification of PLA signals in cells expressing Flag-NOD1 and the indicated SSH1 construct. The graph shows the mean ± SEM of two independent experiments with more than 850 cells analyzed per sample and experiment. (D) Quantification of IL-8 in the supernatants of the cells analyzed in Fig. 4 panel F. Mean + S.D. of triplicates is shown. Related to Figure 4.
(PDF)

Figure S7 **SSH1 does not affect the sub-cellular localization of NOD1 and NOD2.** (A) Indirect immunofluorescence micrographs of HeLa cells transiently transfected with Flag-tagged NOD1 or NOD2. The cells were treated with non-targeting siRNA or a SSH1 specific siRNA where indicated. (B) Knock-down efficiency is shown by immunoblotting. Probing for tubulin served as loading control.
(PDF)

Figure S8 **SSH1 phosphatase activity is involved in Nod1 signaling.** (A) TNF-induced NF-κB activity (filled bars) measured by luciferase reporter assays in HEK293T cells for 6 h with the ROCK inhibitors Y-27632 and Glycyl-H1152. The effect on basal NF-κB activity is shown by open bars. Values are mean + S.D. (n = 3). * p<0.05, ** p<0.005 (two-sided students t-test, compared to mock treated cells “0”). (B) Over expression of SSH1 in HEK293T cells. NF-κB activity measured by luciferase reporter assays. Values are mean + S.D. (n = 3). (C) Effect of the expression of a phosphatase-dead mutant (C393S) of SSH1 on NOD1-mediated NF-κB activity measured by luciferase reporter assays in HEK293T cells. TriDAP induced cells are shown as filled bars, untreated cells only expressing Nod1 are shown as open bars. Values are mean + S.D. (n = 3). Related to Figure 5.
(PDF)

Table S1 **Screen data.** (A) Gene-based list of the inhibiting hits from the HEK293T siRNA-screen. Genes are ranked according to the logP-values derived by applying the RSA-algorithm to the median Z-scores of the four individual siRNAs per gene. (B) Gene-based list of the hits validated in HEK293T cells. Genes are ranked according to the median Z-score of the strongest siRNA. Genes specifically involved in TriDAP-induced NF-κB activation are shown in bold. (C) siRNA-based list of the data obtained from the three secondary screens validation and counter screen using HEK293T cells, as well as the THP1 validation screen.
(XLSX)
