# Supplementary figures and images for: The Cofilin Phosphatase Slingshot Homolog 1 (SSH1) Links NOD1 Signaling to Actin Remodeling
Source: PLoS Pathog. 2014 Sep 4;10(9):e1004351. doi: 10.1371/journal.ppat.1004351 (PMC4154870; doi:10.1371/journal.ppat.1004351)

A

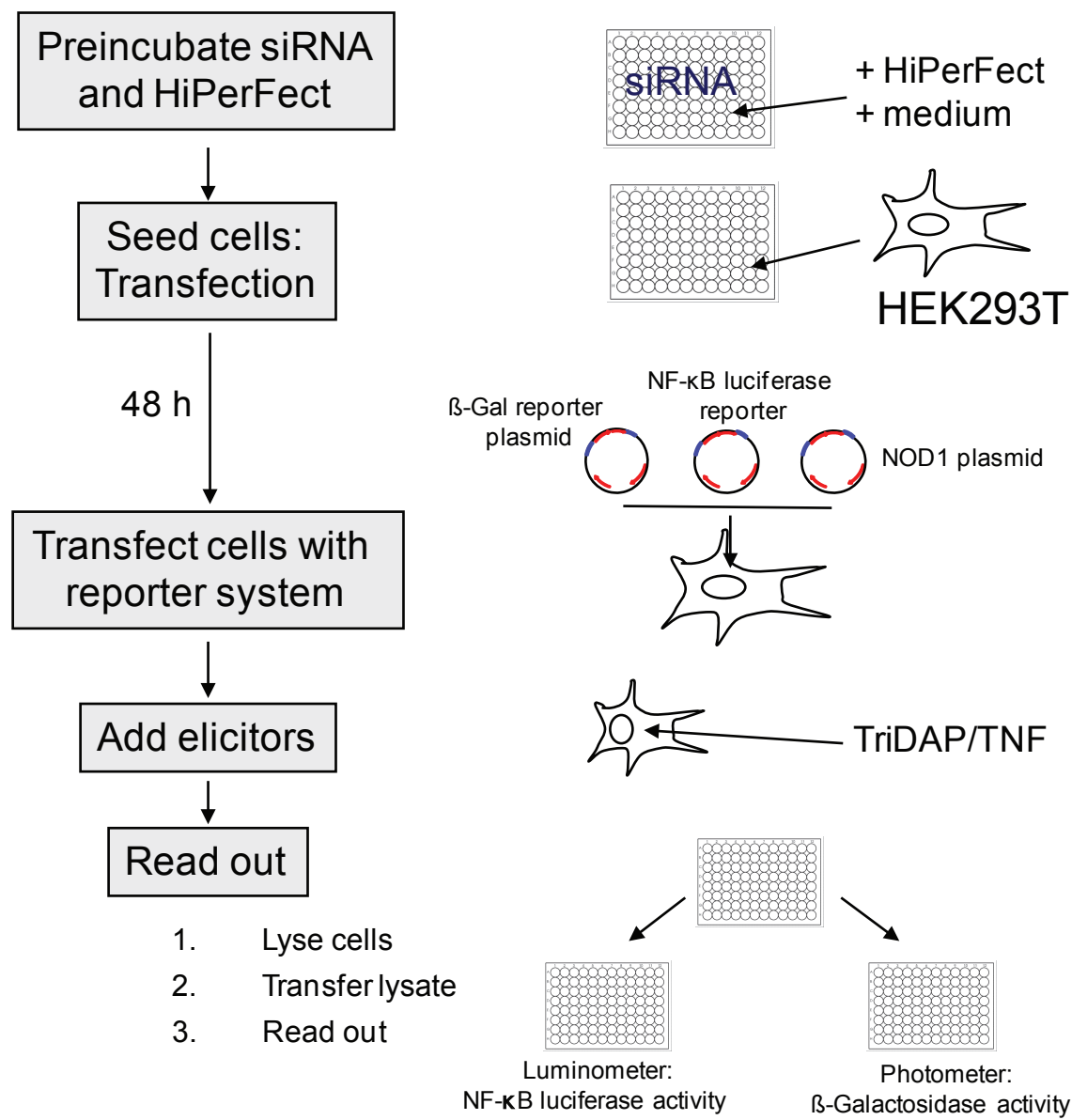

B

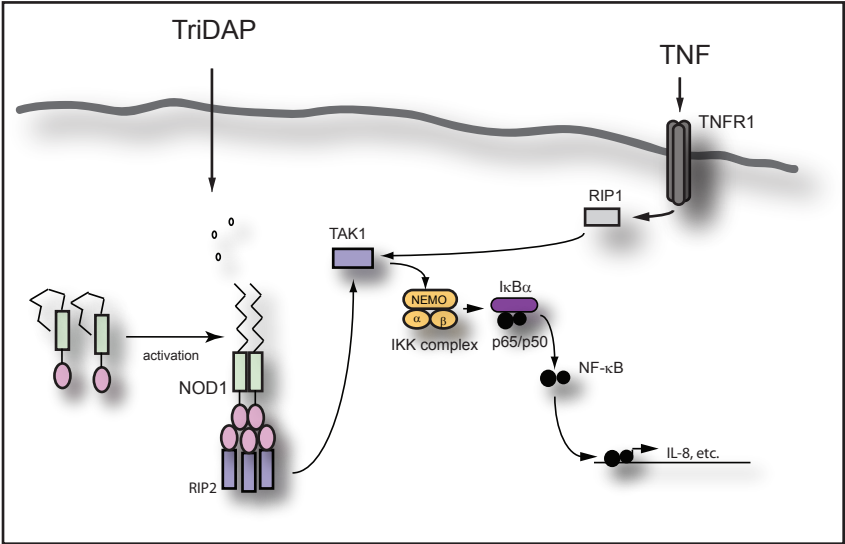

Supplement: Figure S1 — Flow-chart and graphical illustration of the basic screening protocol used in HEK293T cells. (A) Layout of the screening procedure. (B) Schematic representation of the NOD1 signaling pathway. Related to Figure 1. (PDF) [file ppat.1004351.s001.pdf]

A

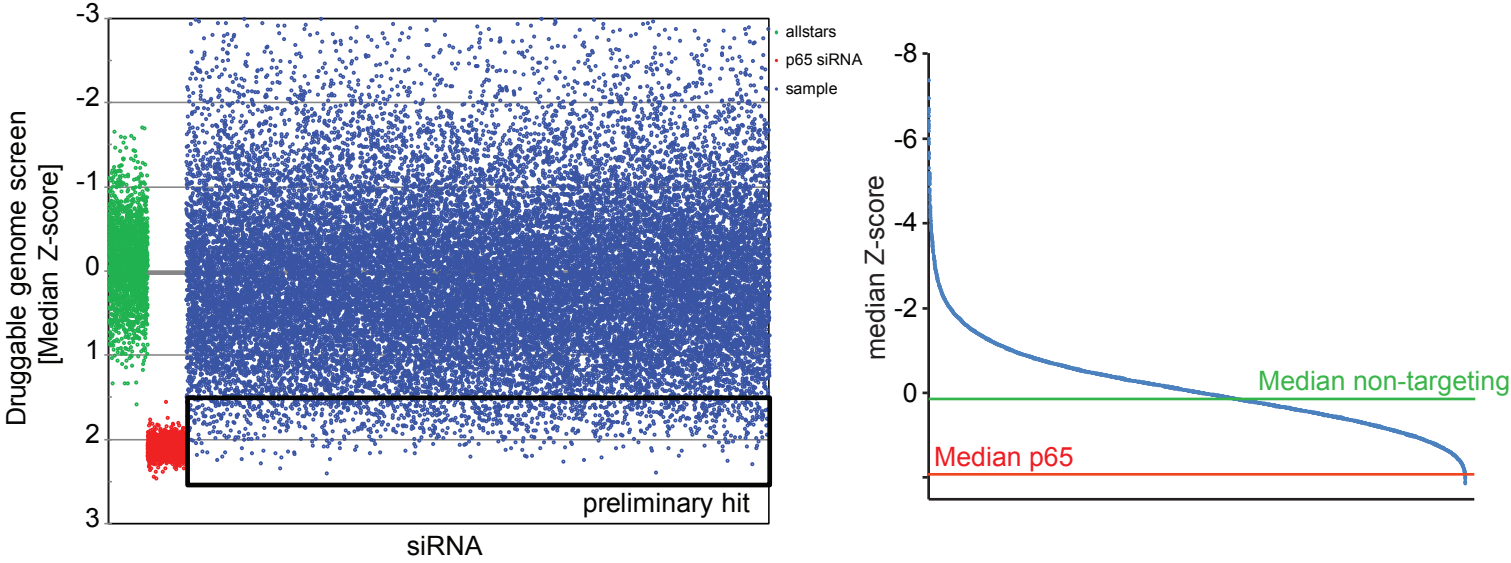

B

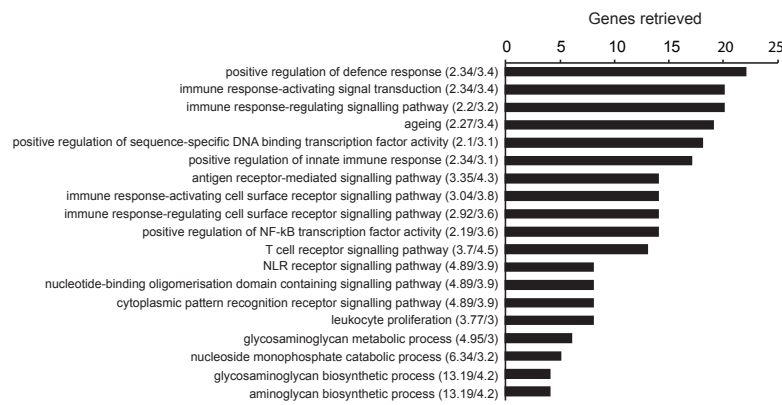

C

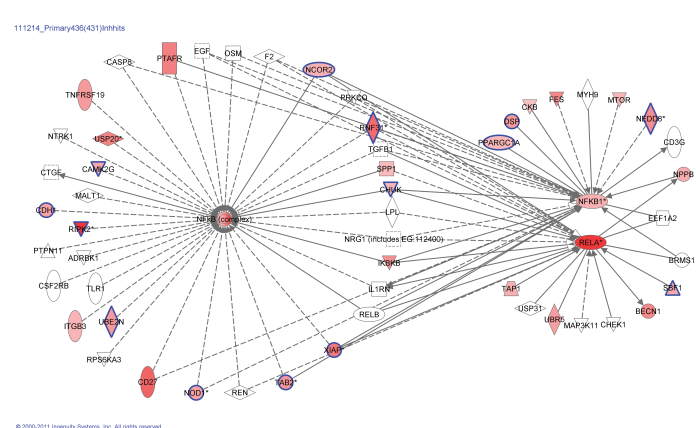

D

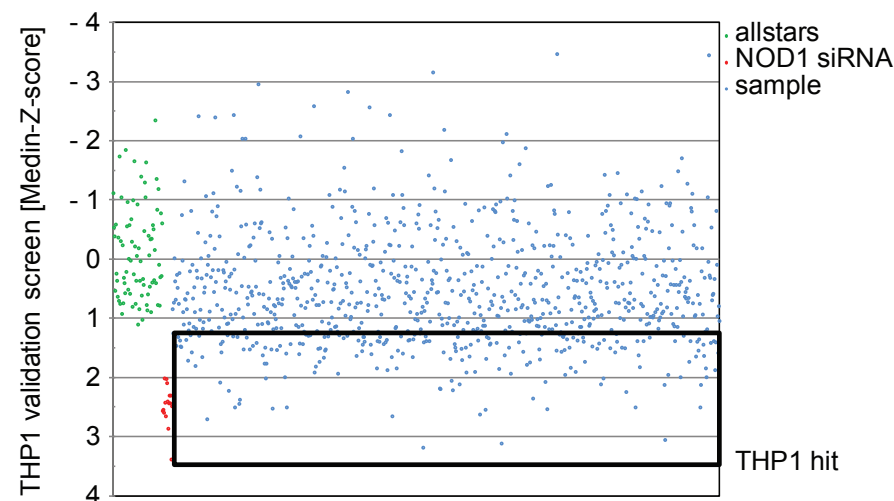

E

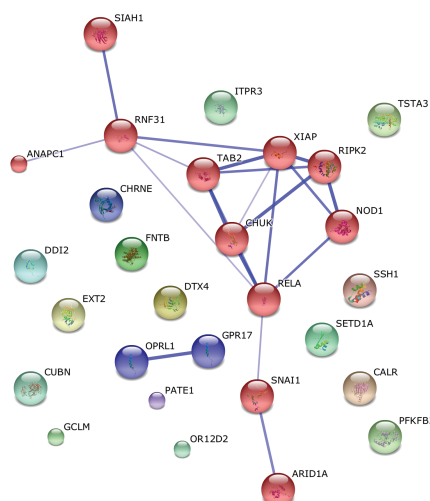

Supplement: Figure S2 — Analysis of the screen data. (A) Distribution of the non-targeting controls (green), positive control (p65, orange) and all tested siRNAs (blue). Left panel: Dot-plot. Right panel: Ranked presentation of all hits. (B) Gene ontology (GO) enrichment of the 435 preliminary hit genes retrieved by the primary screen compared to the druggable-genome library (background). Number of hit genes associated with enriched terms according to the GO of biological processes. Enrichment factors and negative log(P-values) are indicated in brackets. (C) Known regulators of NF-κB retrieved by the screens. Ingenuity Pathway analysis of 56 (out of 435) preliminary inhibiting screen hits already known to be involved in NF-κB regulation. Among them, 28 could be validated in the HEK validation screen (dark red: strong hit; light red: weaker hit; white: not validated). A sub group of 14 were not influencing TNF-α-induced NF-κB activation (blue borders). (D) Distribution of the siRNAs in the THP1-blue validation screen. Each dot represents the median Z-score of the experimental replicates after NPI-normalisation (Normalized Percent Inhibition) to the plate internal NOD1-controls. A positive value represents an inhibitory effect of the respective siRNA on NF-κB activation. THP1 hit-siRNAs are defined as exceeding 1.5 S.D. of CTRL from the median of the CTRL siRNAs. Z-scores were normalized to control siRNAs set to 0. CTRL: non-targeting controls. (E) STRING database analysis of the 28 hits from the THP1 screen. Genes, where the NPI values of both siRNAs exceeded 1.5 S.D. of CTRL from the median of CTRL were regarded as validated. Stronger associations are represented by thicker blue lines. Genes were clustered according to the Marcov cluster (MCL) algorithm. Related to Figure 1. (PDF) [file ppat.1004351.s002.pdf]

# Bielig et al. , Figure S3

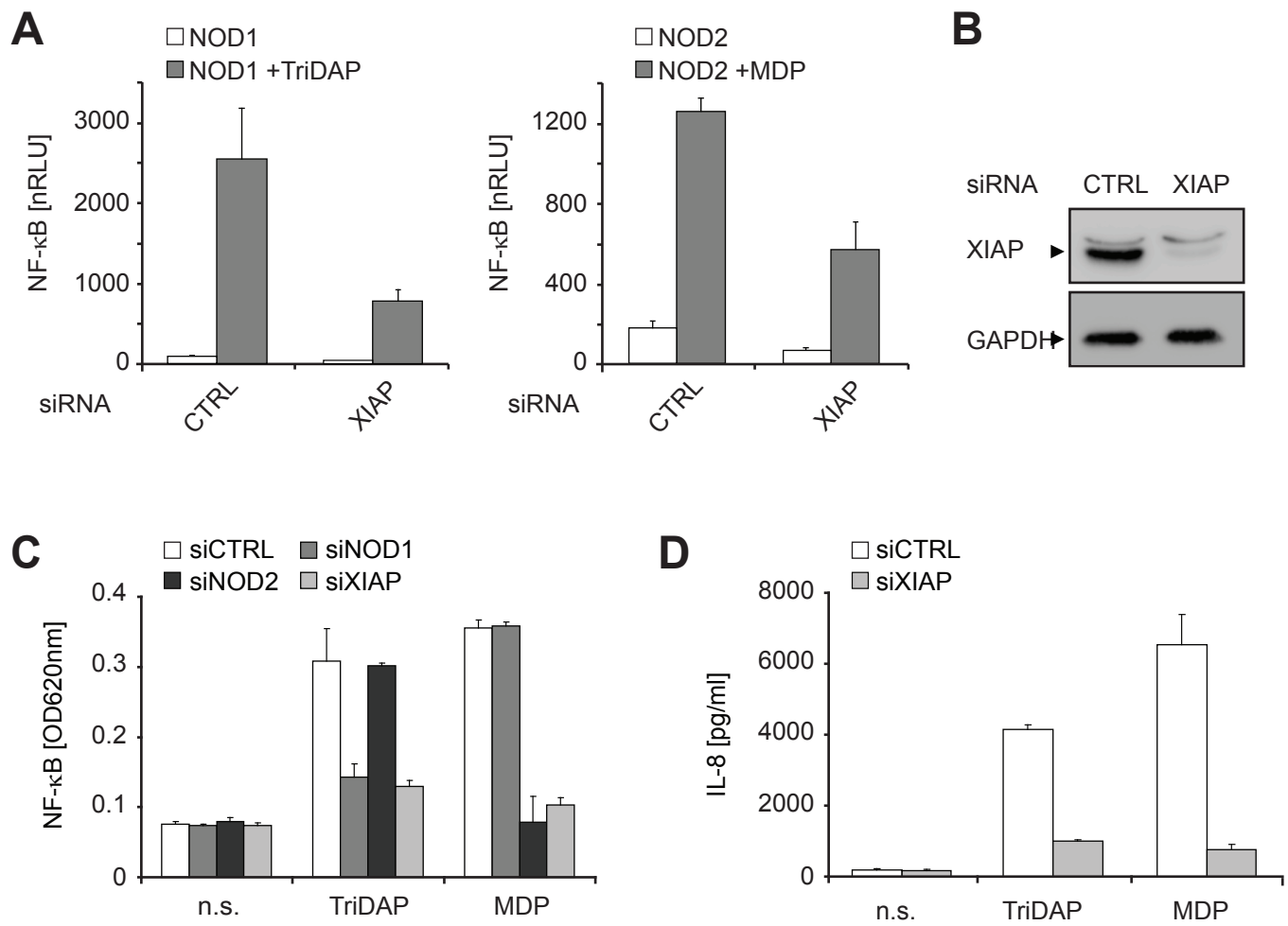

Supplement: Figure S3 — Validation of XIAP as essential component of the NOD1 signaling pathway. (A) HEK293T cells were transfected with CTRL or XIAP siRNA and incubated for 48 h. Subsequently, cells were transfected with the NF-κB luciferase RPS- containing NOD1 (left panel) or NOD2 (right panel) expression plasmids and stimulated with TriDAP (0.5 µM) or MDP (50 nM), respectively. After 16 h, cells were lysed, luciferase activation was determined and normalized with the β-galactosidase values (nRLU). Values are mean + S.D. (n = 3). (B) Confirmation of the XIAP knock-down on protein level. HEK293T cells were transfected with CTRL or XIAP siRNA. After 72 h, cells were lysed and XIAP protein levels were determined by Western blot using a XIAP-specific antibody. Detection of GAPDH served as loading control. (C) Differentiated THP1-blue cells were transfected with CTRL, NOD1, NOD2, or XIAP siRNA. Cells were incubated for 72 h and subsequently stimulated with TriDAP or MDP (10 µg/ml each), 16 h later, NF-κB activation was determined by measurement of SEAP activity in the supernatants. (D) In parallel, IL-8 secretion was determined by ELISA. Values are NF-κB activation [OD620] or IL-8 secretion [pg/ml], respectively, mean +SD (CTRL, XIAP: n = 3; NOD1, NOD2: n = 2). Data is representative for at least three independent experiments. Related to Figure 1. (PDF) [file ppat.1004351.s003.pdf]

**A**

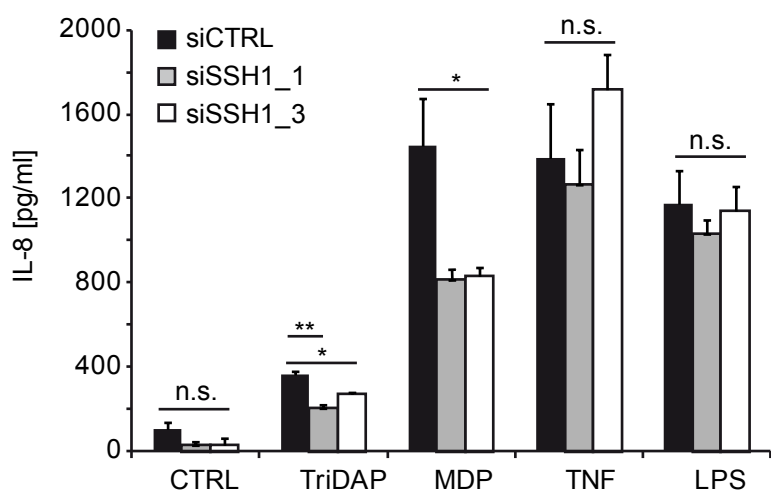

**B**

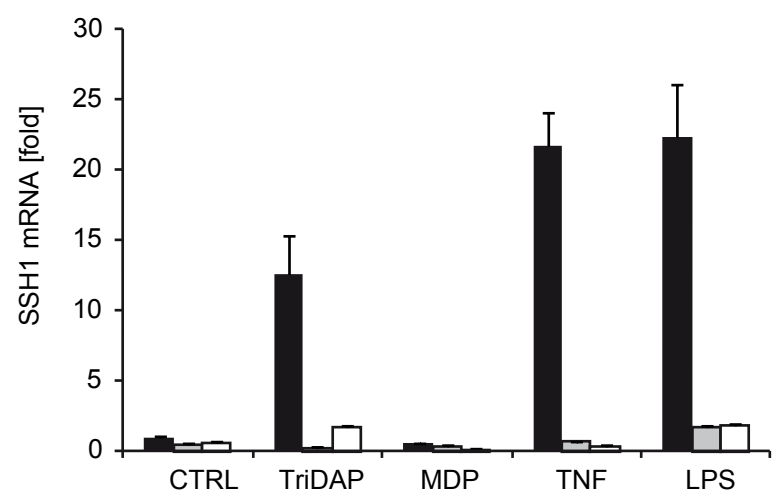

**C**

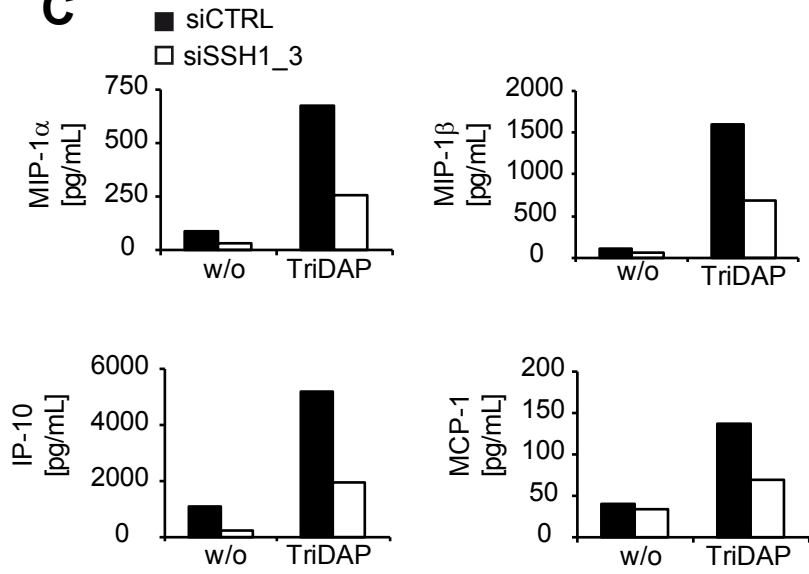

Supplement: Figure S4 — SSH1 knock-down effect in THP1 cells. PMA differentiated THP1 cells were treated for 72 h with a non-targeting (siCTRL) or two SSH1 specific siRNA duplex. (A) IL-8 secretion was measured by ELISA after stimulation for 16 h with TriDAP (10 µg/ml), MDP (10 µg/ml), TNF (0.1 µg/ml), and or LPS (0.05 µg/ml) stimulation. (B) In parallel, SSH1 mRNA levels were determined by qPCR from the same cells. Values are Mean + S.D. (n = 3) representative of at least three independent experiments. * p<0.05. n.s.: not significant. (C) Cytokine release of 20 relevant inflammatory cytokines of the cells in (A) after TriDAP stimulation was analyzed by multiplex bead array. Changes in the cytokine response following SSH1 depletion by siRNA is shown for the proteins with the highest induction. Related to Figure 2. (PDF) [file ppat.1004351.s004.pdf]

Bielig et al., Figure S5

A

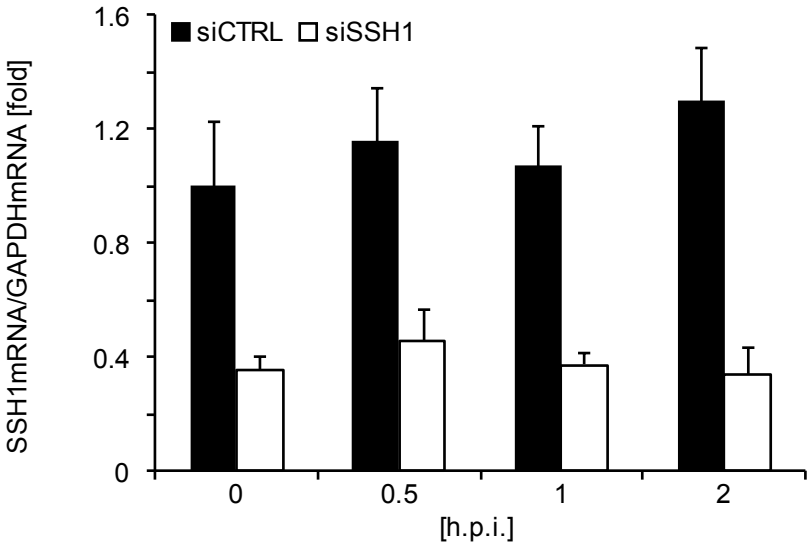

B

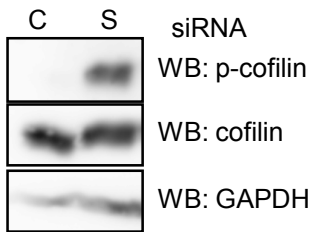

C

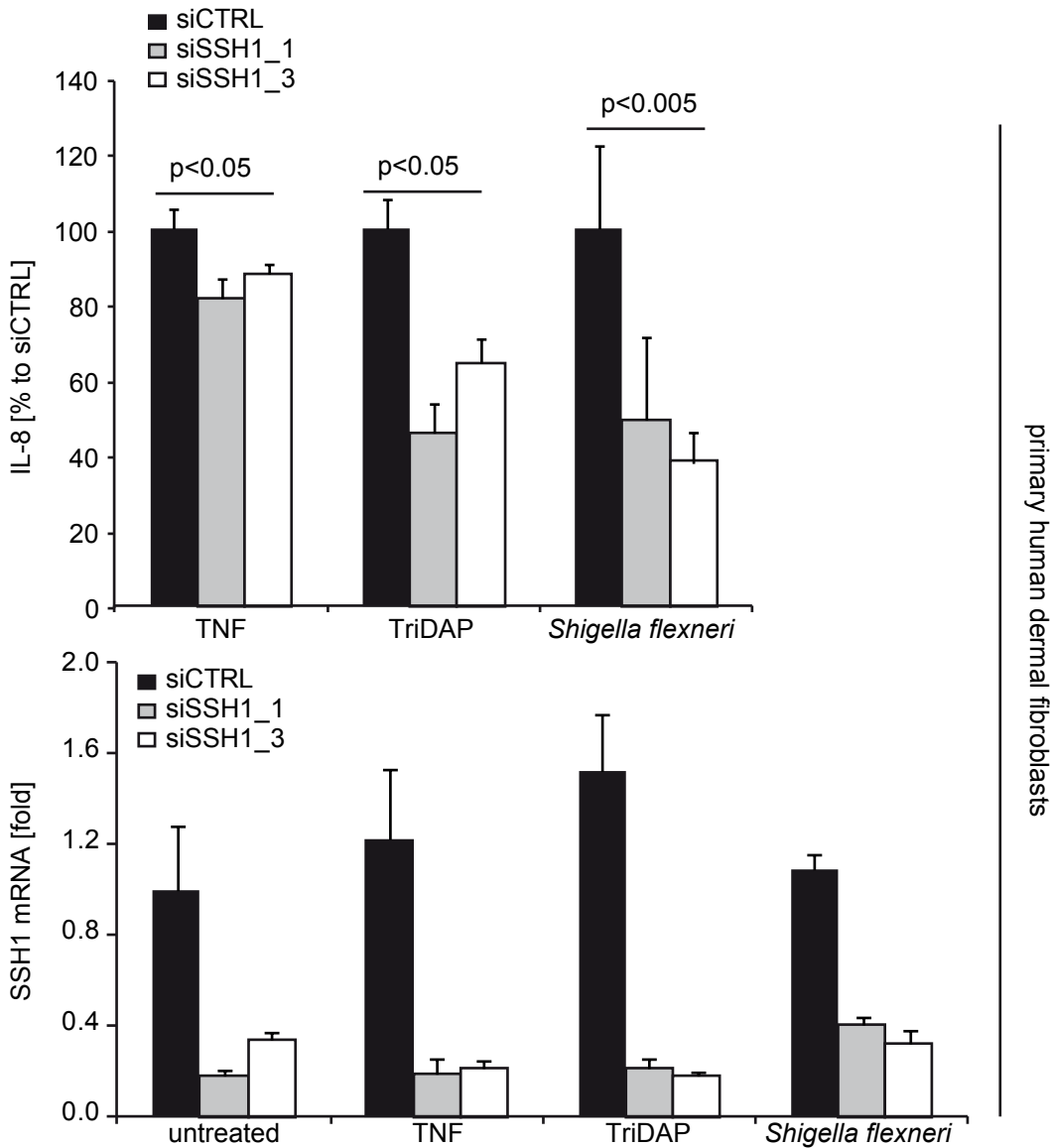

Supplement: Figure S5 — SSH1 knock-down phenotype in HeLa and primary human dermal fibroblasts. (A) qPCR analysis of SSH1 levels in the experiment shown in Figure 3C. (B) Immunoblotting for cofilin S3-phosphorlyation in HEK293T cells after SSH1 knock-down. (C) Primary human dermal fibroblasts of early passages were transfected with the indicated siRNA for 72 h and subsequently treated with TNF, TriDAP, or infected with S.flexneri and IL-8 release was measured 6 h later by ELISA (upper panel). Percent IL-8 level compared to cells treated with the siCTRL SSH1 knock-down is shown by qPCR from the same cells (lower panel). Mean + S.D. (n = 3) representative of experiments from cells of two different donors is shown. Related to Figure 3. (PDF) [file ppat.1004351.s005.pdf]

# Bielig et al., Figure S6

**A**

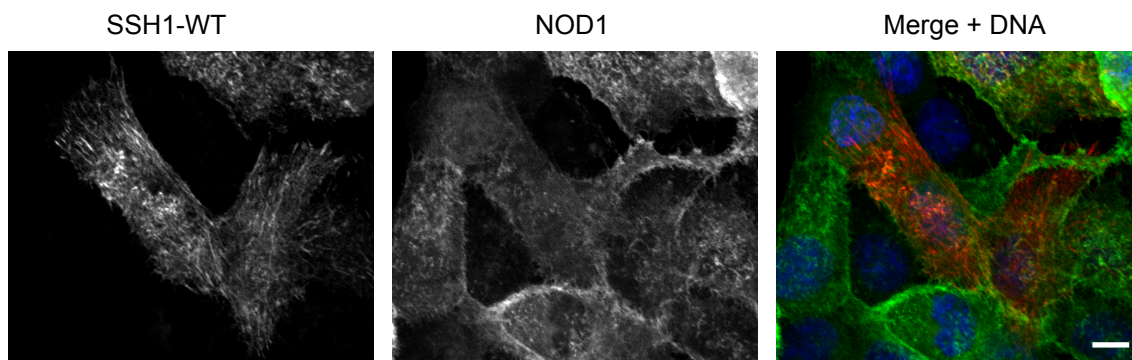

**B**

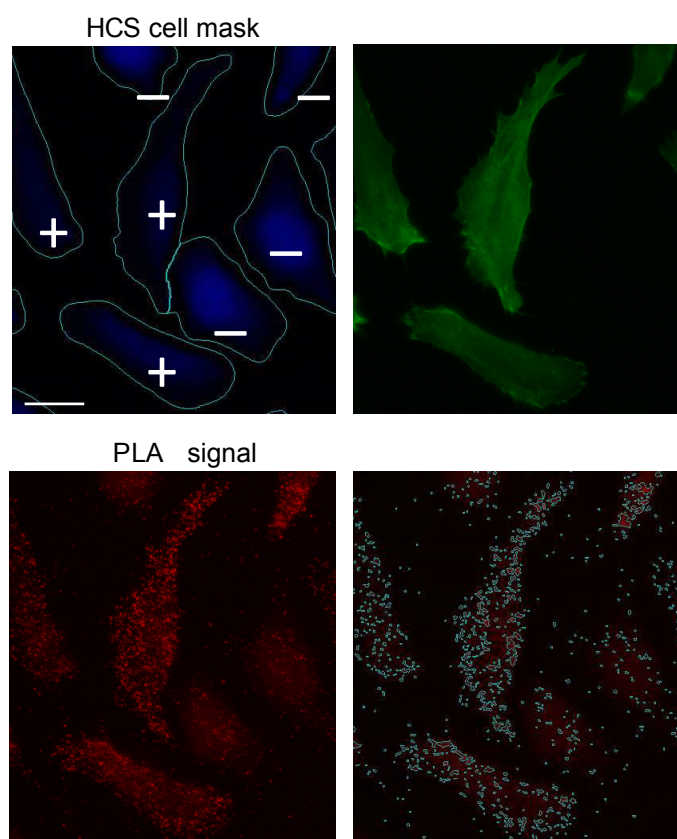

**C**

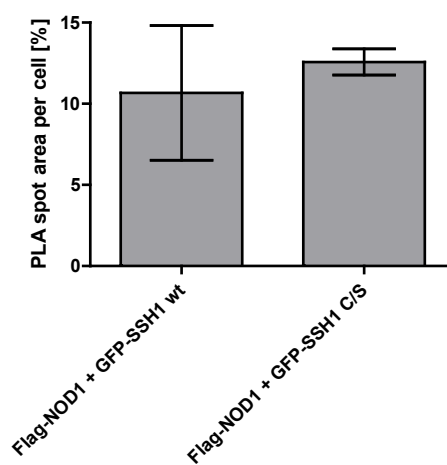

**D**

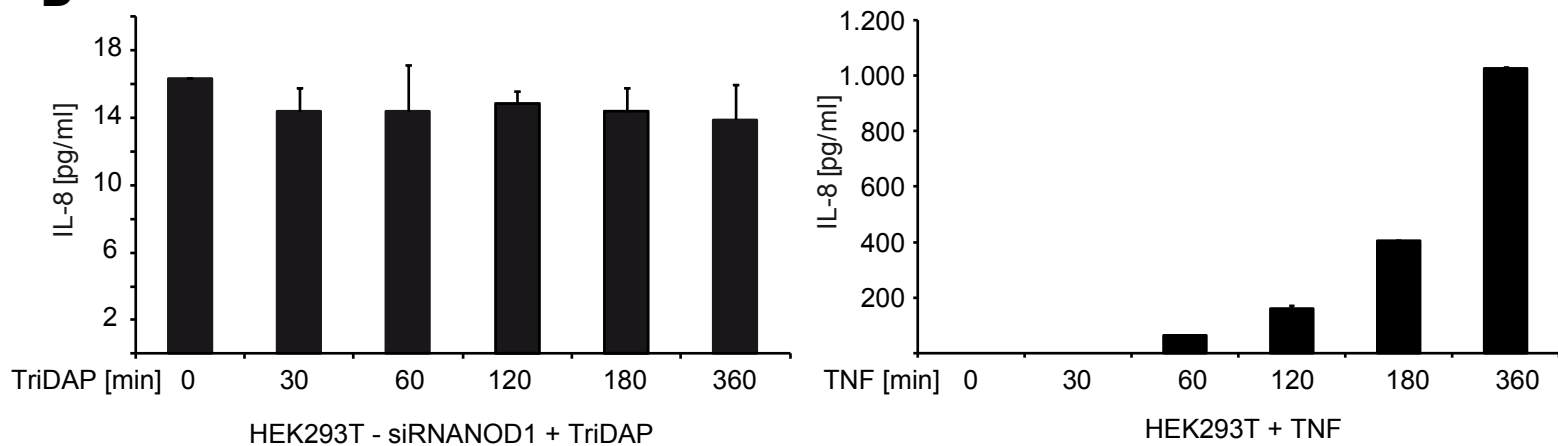

Supplement: Figure S6 — SSH1 co-localizes with Nod1. (A) Indirect immunofluorescence images of HeLa cells expressing GFP-NOD1 and myc-SSH1. NOD1 signal is shown in green in the merge image together with the SSH1 signal in red and DNA staining in blue. (B) HCS/HTS – based PLA spot quantification. Images show cell segmentation with HCS cell mask stain (blue channel). Cell classification in EGFP-SSH1 positive/negative (green channel, indicated in blue channel) and automated PLA-spot detection (red channel). Scale bar 25 µm. (C) Quantification of PLA signals in cells expressing Flag-NOD1 and the indicated SSH1 construct. The graph shows the mean ± SEM of two independent experiments with more than 850 cells analyzed per sample and experiment. (D) Quantification of IL-8 in the supernatants of the cells analyzed in Fig. 4 panel F. Mean + S.D. of triplicates is shown. Related to Figure 4. (PDF) [file ppat.1004351.s006.pdf]

Bielig et al., Figure S7

A

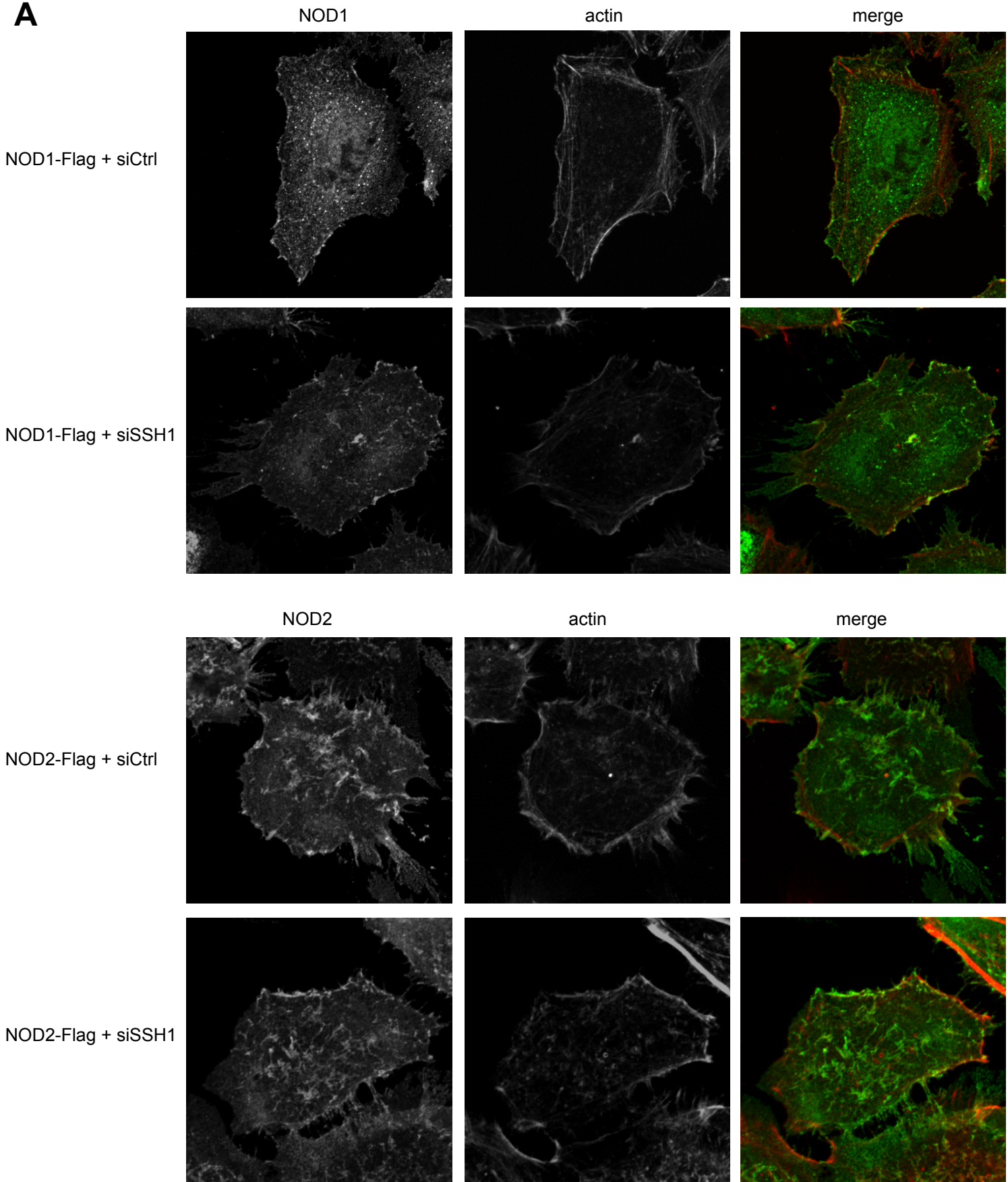

B

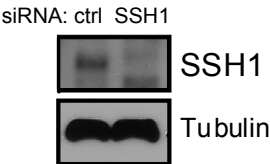

Supplement: Figure S7 — SSH1 does not affect the sub-cellular localization of NOD1 and NOD2. (A) Indirect immunofluorescence micrographs of HeLa cells transiently transfected with Flag-tagged NOD1 or NOD2. The cells were treated with non-targeting siRNA or a SSH1 specific siRNA where indicated. (B) Knock-down efficiency is shown by immunoblotting. Probing for tubulin served as loading control. (PDF) [file ppat.1004351.s007.pdf]

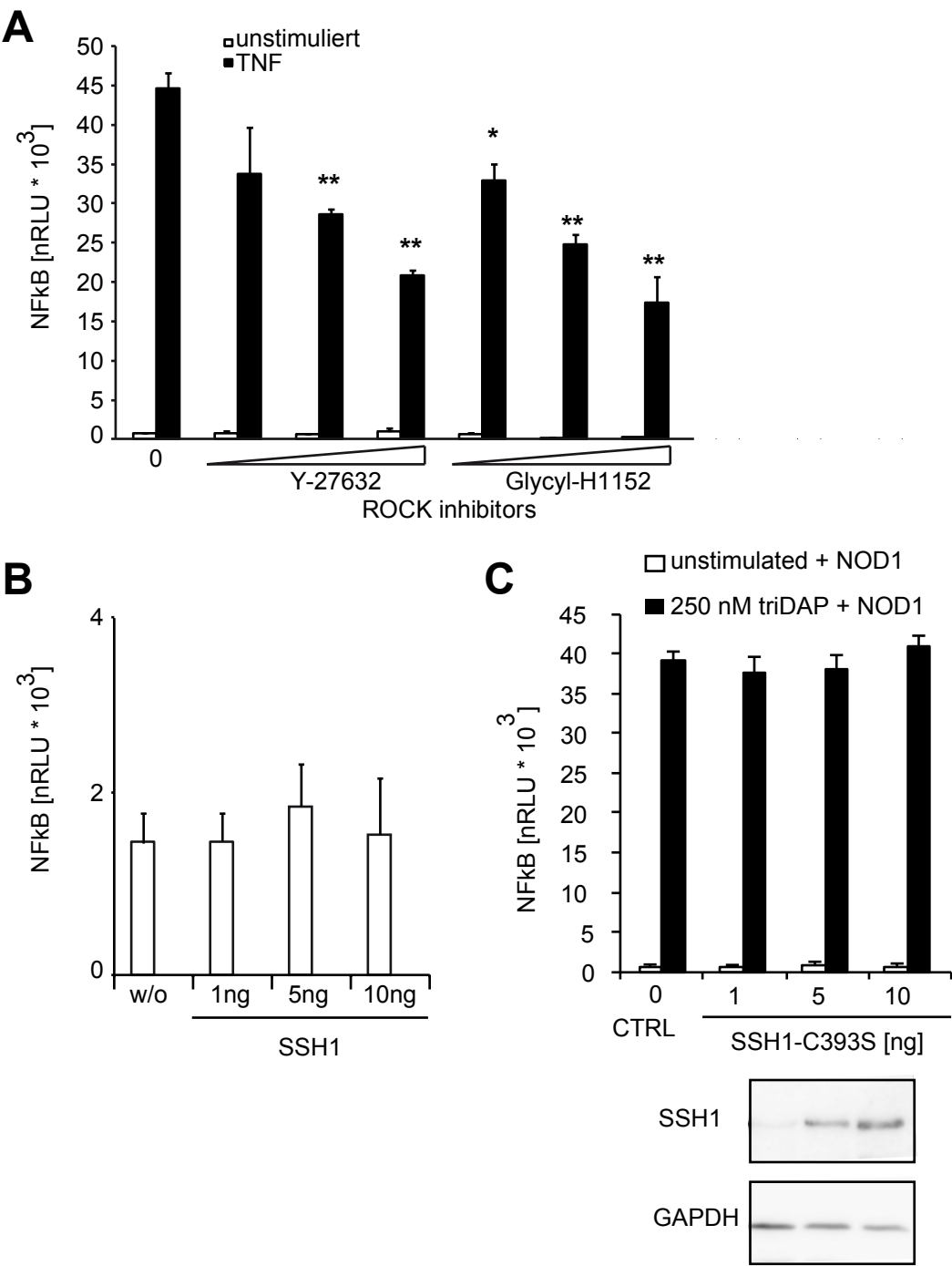

Supplement: Figure S8 — SSH1 phosphatase activity is involved in Nod1 signaling. (A) TNF-induced NF-κB activity (filled bars) measured by luciferase reporter assays in HEK293T cells for 6 h with the ROCK inhibitors Y-27632 and Glycyl-H1152. The effect on basal NF-κB activity is shown by open bars. Values are mean + S.D. (n = 3). * p<0.05, ** p<0.005 (two-sided students t-test, compared to mock treated cells “0”). (B) Over expression of SSH1 in HEK293T cells. NF-κB activity measured by luciferase reporter assays. Values are mean + S.D. (n = 3). (C) Effect of the expression of a phosphatase-dead mutant (C393S) of SSH1 on NOD1-mediated NF-κB activity measured by luciferase reporter assays in HEK293T cells. TriDAP induced cells are shown as filled bars, untreated cells only expressing Nod1 are shown as open bars. Values are mean + S.D. (n = 3). Related to Figure 5. (PDF) [file ppat.1004351.s008.pdf]
